# Artificial intelligence analysis of the impact of fibrosis in arrhythmogenesis and drug response

**DOI:** 10.3389/fphys.2022.1025430

**Published:** 2022-10-12

**Authors:** Ana María Sánchez de la Nava, Lidia Gómez-Cid, Alonso Domínguez-Sobrino, Francisco Fernández-Avilés, Omer Berenfeld, Felipe Atienza

**Affiliations:** ^1^ Department of Cardiology, Hospital General Universitario Gregorio Marañón, Instituto de Investigación Sanitaria Gregorio Marañón (IISGM), Madrid, Spain; ^2^ Centro de Investigación Biomédica en Red de Enfermedades Cardiovasculares (CIBERCV), Madrid, Spain; ^3^ Universidad Complutense de Madrid, Madrid, Spain; ^4^ Center for Arrhythmia Research, University of Michigan, Ann Arbor, MI, United States

**Keywords:** atrial fibrillation, cardiac fibrosis, machine learning, population of models, support vector machines

## Abstract

**Background:** Cardiac fibrosis has been identified as a major factor in conduction alterations leading to atrial arrhythmias and modification of drug treatment response.

**Objective:** To perform an *in silico* proof-of-concept study of Artificial Intelligence (AI) ability to identify susceptibility for conduction blocks in simulations on a population of models with diffused fibrotic atrial tissue and anti-arrhythmic drugs.

**Methods:** Activity in 2D cardiac tissue planes were simulated on a population of variable electrophysiological and anatomical profiles using the Koivumaki model for the atrial cardiomyocytes and the Maleckar model for the diffused fibroblasts (0%, 5% and 10% fibrosis area). Tissue sheets were of 2 cm side and the effect of amiodarone, dofetilide and sotalol was simulated to assess the conduction of the electrical impulse across the planes. Four different AI algorithms (Quadratic Support Vector Machine, QSVM, Cubic Support Vector Machine, CSVM, decision trees, DT, and K-Nearest Neighbors, KNN) were evaluated in predicting conduction of a stimulated electrical impulse.

**Results:** Overall, fibrosis implementation lowered conduction velocity (CV) for the conducting profiles (0% fibrosis: 67.52 ± 7.3 cm/s; 5%: 58.81 ± 14.04 cm/s; 10%: 57.56 ± 14.78 cm/s; *p* < 0.001) in combination with a reduced 90% action potential duration (0% fibrosis: 187.77 ± 37.62 ms; 5%: 93.29 ± 82.69 ms; 10%: 106.37 ± 85.15 ms; *p* < 0.001) and peak membrane potential (0% fibrosis: 89.16 ± 16.01 mV; 5%: 70.06 ± 17.08 mV; 10%: 82.21 ± 19.90 mV; *p* < 0.001). When the antiarrhythmic drugs were present, a total block was observed in most of the profiles. In those profiles in which electrical conduction was preserved, a decrease in CV was observed when simulations were performed in the 0% fibrosis tissue patch (Amiodarone ΔCV: −3.59 ± 1.52 cm/s; Dofetilide ΔCV: −13.43 ± 4.07 cm/s; Sotalol ΔCV: −0.023 ± 0.24 cm/s). This effect was preserved for amiodarone in the 5% fibrosis patch (Amiodarone ΔCV: −4.96 ± 2.15 cm/s; Dofetilide ΔCV: 0.14 ± 1.87 cm/s; Sotalol ΔCV: 0.30 ± 4.69 cm/s). 10% fibrosis simulations showed that part of the profiles increased CV while others showed a decrease in this variable (Amiodarone ΔCV: 0.62 ± 9.56 cm/s; Dofetilide ΔCV: 0.05 ± 1.16 cm/s; Sotalol ΔCV: 0.22 ± 1.39 cm/s). Finally, when the AI algorithms were tested for predicting conduction on input of variables from the population of modelled, Cubic SVM showed the best performance with AUC = 0.95.

**Conclusion:** *In silico* proof-of-concept study demonstrates that fibrosis can alter the expected behavior of antiarrhythmic drugs in a minority of atrial population models and AI can assist in revealing the profiles that will respond differently.

## Introduction

Cardiac fibrosis has been identified as a major pro-arrhythmic factor associated with impaired electrical conductance and reentries. Two different mechanisms have been proposed to underlie the possible reentrant patterns: reentry due to an anatomical obstacle or functional reentry (Yeo et al., 2017). Contrary to cardiomyocytes, fibroblasts are non-excitable cells. Therefore, the increased presence of cardiac fibrosis or fibroblasts can form areas of reduced conduction velocity in the anatomy of the cardiac tissue that increase the susceptibility to initiation and maintenance of cardiac arrhythmias (Krul et al., 2015). Overall, the combination of cardiac fibrosis in the presence of atrial fibrillation (AF), represents a synergetic proarrhythmic framework relative to patients suffering AF without cardiac fibrosis (Marrouche et al., 2014). As the fibrotic AF scenario is biologically and electrophysiologically more complex than AF alone, the effects of therapies such as antiarrhythmic drugs (AADs) would benefit from evaluation on relevant population models.

AADs are a group of pharmacological compounds that modify the rhythm of the heart by blocking one or several ionic channels controlling the electrical activation of the excitable cells of the heart (Sanguinetti and Bennett, 2003). While AADs may be particularly helpful in the treatment of AF caused by functional reentries or reentries caused by hyper-excitability of the cardiac tissue, in the presence of fibrosis and low conduction velocity of the cardiac tissue, AADs may cause undesired effects and actually exacerbate proarrhythmic factors. Among the most common AADs used for AF treatment in clinical practice we can find amiodarone, dofetilide, sotalol, flecainide or verapamil (Zimetbaum, 2012). The use of antiarrhythmic drugs in scenarios with fibrosis has been previously described (Zimetbaum, 2012; Saljic and Heijman, 2022) as proarrhythmic due to heterogeneous conduction (Cox et al., 1995), however a systematic evaluation of arrhythmogenicity produced by the many possible combinations of electrophysiological and fibrosis factors in AF is lacking.

Artificial Intelligence (AI), on the other hand, has been rapidly incorporated in biomedical applications to analyze and detect possible patterns or associations in large datasets that enable identification of mechanistic relationships and predict outcomes (Sánchez de la Nava et al., 2021b). Previous studies using population of models have underscored the importance of different ionic profiles on drug effect (Sanchez de la Nava et al., 2021a) and the potential proarrhythmic role of fibrosis (Kazbanov et al., 2016). Therefore, in this study we explore the effects of different AADs on the electrical impulse propagation using a population of different atrial models with diffused fibrosis distribution, and test the ability of AI algorithms to predict conduction patterns relevant to initiation and maintenance of AF.

## Materials and methods

### Cellular models and tissue connection

Two different models were implemented to simulate cardiac tissue with different levels of fibrosis. The cardiomyocyte model was the Koivumaki Model with Skibsbye modifications that mimick AF remodelling (Skibsbye et al., 2016) and described the electrophysiological behavior of human atrial cardiomyocyte, as previously presented by our group (Sanchez de la Nava et al., 2021a). The fibroblast model implemented in this study corresponded to the Maleckar model (Maleckar et al., 2009) that emulates the behavior of active fibroblasts along the atrial tissue based on the model developed by MacCannell (MacCannell et al., 2007). In total, four different currents were modeled, including the time and voltage dependent fibroblast K^+^ current (I_kv_), the time-independent inward-rectifier current (I_K1_), the fibroblast Na^+^-K^+^ pump current (I_NaK_) and the fibroblast background Na^+^ current (I_Na-b_) and adapted to atrial electrophysiology. Then, heterogeneous coupling was simulated through Na^+^ and K^+^ movement through gap junctions (I_Gap_), which assumed these two species as independent. This I_Gap_ current depends on the transmembrane voltage difference between the cardiomyocyte and the neighboring fibroblast, and on the gap conductance which was fixed (G_gap_ = 0.5 nS, taking into account both G_gap-Na_ and G_gap-K_). This I_Gap_ current contributed to the term I_ion_ in the equation below, when a communication among a cardiomyocyte and a fibroblast was simulated. Initial conditions were applied according to the stablished protocol as follows: resting membrane potential (RMP) was set to -47.75 mV for fibroblasts and -79.83 mV for cardiomyocytes, the capacitance of the cell membrane (C_m_) was set to 6.3 pF for fibroblasts and 66 pF for cardiomyocytes, [K^+^]_i_ and [Na^+^]_i_ were set to 129.43 mM and 8.5547 mM, respectively. Main parameters used for the cardiomyocyte and the fibroblast model are shown in Supplementary Tables S1,2.

Diffused fibrosis was modeled using planes of the same size where the fibroblasts were randomly located considering two different distributions that differed in the percentage: 0%, 5% and 10% of fibrotic cells, percentages similar to those in other studies (Kazbanov et al., 2016; Palacio et al., 2021). Simulation protocols were performed on 2D planes mimicking a sheet of cardiac tissue of 200 x 200 nodes (2 cm side plane) that constitute around 20–25% of the total area of a human atria (Sachse, 2004). No-flux boundary condition was implemented at the edges. To connect the cells within the plane, the monodomain reaction-diffusion equation was implemented, assuming that tissue behaves as a functional syncytium where membrane voltage is propagated smoothly (Clayton and Panfilov, 2008):

$$\frac{\partial V_{m}}{\partial t}=\nabla \cdot \left(D\nabla V_{m}\right)-\frac{I_{ion}+I_{applied}}{C_{m}}$$

Where V_m_ is the transmembrane potential, *t* is the time, ∇ corresponds to the gradient operator and *D* a diffusion coefficient with units distance^2^ time^−1^, *I*_*ion*_ is the sum of all modeled transmembrane ionic currents, *I*_*applied*_ is the externally applied stimulus current, and *C*_*m*_ is the capacitance of the cell membrane. By using this monodomain simplification, the tissue is considered to have an unlimited extracellular medium, so the extracellular resistivity can be neglected. The extracellular medium is isopotential and equal to zero for simplicity. Consequently, the membrane potential is the same as the intracellular potential. Planes were fully connected not including structures such as the pulmonary veins.

### Electrophysiological variability: Population of models approach

The equations included in the aforementioned cellular models depend on constants such as channel conductivities, ionic concentrations and diffusion factors. A population of models allows mathematical computations to consider variations of the initial variables introduced in the model to account for the genetic variability present in real patients. In this case, a population of 127 ionic profiles was used including the variability present in a set of human data (Liberos et al., 2016; Simon et al., 2017).

Briefly, experimental data to calibrate the population was obtained by patch clamp techniques on myocardium atrial tissue of 149 AF patients (Sánchez et al., 2014). From these experiments, nine specific electrophysiological variables were measured to account for variation: g_Na_, I_NaK_, g_K1_, g_CaL_, g_Kur_, I_KCa_, diffusion (D), extracellular potassium concentration and extracellular sodium concentration. Action potential biomarkers were measured (Sánchez et al., 2014) and later used as reference (Supplementary Tables S3) to ensure that simulations were within physiological ranges. From the electrophysiological variables measured, Latin Hypercubic Sampling (LHS) was run to amplify the set of combinations to a final number of 500. These 500 combinations were included in an *in silico* tissue model of 8x256 cells in which electrophysiological properties were measured (Simon et al., 2017), Action potential biomarkers were evaluated to ensure that simulations were within physiological ranges and only the combinations within these ranges (Supplementary Tables S3) were included in the final population. A complete description of the calibration of this population can be consulted in previous publications of the group (Simon et al., 2017; Sanchez de la Nava et al., 2021a). The modification of the values (in percentage with respect to the baseline cardiomyocyte model) for each parameter in the different profiles in the population of models is shown in Supplementary Table S1, and the distribution of the range of parameter variation (-50% to +100%) is shown in Supplementary Figure S1.

### Drug implementation

All antiarrhythmic drugs were evaluated in the electrophysiological population of models in order to characterize the effect according to the fibrosis percentage. The Single Pore Channel Model was implemented to analyze the effect of three different drugs (amiodarone, dofetilide and sotalol). This model inhibits the current by decreasing the conductance of the channel as described in the following equation:

$$G_{i}=G_{0}\cdot \;\frac{1}{1+\frac{{\left[C_{d}\right]}_{i}}{{IC}_{50}}}$$

Where G_0_ corresponds to the initial conductance of the channel, G_i_ corresponds to the final conductance of the channel, [C_d_]_i_ is the concentration of the drug and IC_50_ is the concentration of the drug that reduces by 50% the channel current. The values of IC_50_ and drug concentration can be observed in Table 1, and were obtained from (Patel et al., 2019).

**TABLE 1:** Constants for drug modelling.

Drug	[C_d_]i (µM)	IC50 IKr (µM)	IC50 ICaL (µM)	IC50 INa (µM)
Amiodarone	0.8	0.9	1.3	4.6
Dofetilide	0.005	0.002	0.006	0.006
Sotalol	86.3	2100	2100	2.1

### Simulation protocols

Simulations were performed by implementing partial differential equations for the transmembrane potential models of all cells computed with a time step of 1 µs in the forward Euler scheme using in-house C++ code with CUDA parallelization solved on an NVIDIA TESLA C2057 GPU (NVIDIA Corporation, Santa Clara, CA). The Rush-Larsen scheme (Rush and Larsen, 1978) was used for gating variables in cell models of the form

$$\frac{dw_{i}}{dt}=\alpha_{w_{i}}\left(1-w_{i}\right)-\beta_{w_{i}}\;w_{i}$$

where w is the corresponding gating variable and $\alpha_{w_{i}}$ = $\alpha_{w_{i}}\left(V\right)$ and $\beta_{w_{i}}$ = $\beta_{w_{i}}\left(V\right)$ are the voltage dependent rate constants. The Rush-Larsen method provides a stable temporal solution for the gating variables by relaying on the exact exponential solution implemented in the following expression (Perego and Veneziani, 2009) for each cell:

$$w_{i+1}^{j}=e^{a_{w_{i}}\left(V\right)h}\left(w_{i}^{j}+\frac{b_{w_{i}}\left(V\right)}{a_{w_{i}}\left(V\right)}\right)-\frac{b_{w_{i}}\left(V\right)}{a_{w_{i}}\left(V\right)}$$

where $a_{w_{i}}=-\left(\alpha_{w_{i}}+\beta_{w_{i}}\right)$ , $b_{i}=\alpha_{w_{i}}$ , *j* corresponds to each individual cell, and *h* is the time step for the forward time index *i* integration.

Planes were simulated for a total of four impulses (S1) of magnitude 4000 pA/pF and duration 3 ms applied at the left edge of the planes in 200 cells at a frequency of 1 Hz. From the complete set of simulations, four different biomarkers were evaluated considering the last two S1 pulses: the Action Potential Duration at 90% repolarization (APD90, measured in ms), the conduction velocity (CV, measured in cm/s), the resting membrane potential (RMP, measured in mV) and peak voltage value (Peak, measured in mV). APD90, RMP and Peak were calculated by averaging the values of all the cardiomyocytes present in the plane. Cell activation time was marked at the time of highest voltage time derivative (Simon et al., 2017). For CV propagation measurements, the distance between two points at coordinates (0.5 mm, 10 mm) and (19.5 mm, 10 mm) in the 2D plane was divided by the activation time differences between these points. Absence of conduction was considered when the cell in the second coordinate (right side of the plane) did not activate (depolarize and repolarize) after the application of the last stimulus in the left side of the plane. This included some profiles that failed to repolarize, some profiles that failed to conduct and some profiles that failed to be stimulated. All measurements reported in this study are the average value of each biomarker during the last two S1 pulses. To evaluate the effect of a drug, the difference in value of the aforementioned biomarkers was computed to quantify and increase or decrease relative to a basal value as:

$$∆Biomarker={Biomarker}_{Drug}-{Biomarker}_{Basal}$$

### Artificial intelligence algorithm: Pattern recognition for non-conducting and conducting profiles

A total of 1032 simulations were computed in this study corresponding to all different combinations of the population of models with different fibrosis degree (0% fibrosis, 5% fibrosis and 10% fibrosis) under the effect of different antiarrhythmic drugs (amiodarone, dofetilide and sotalol), simulated in sheets of cardiac tissue.

**FIGURE 1:**
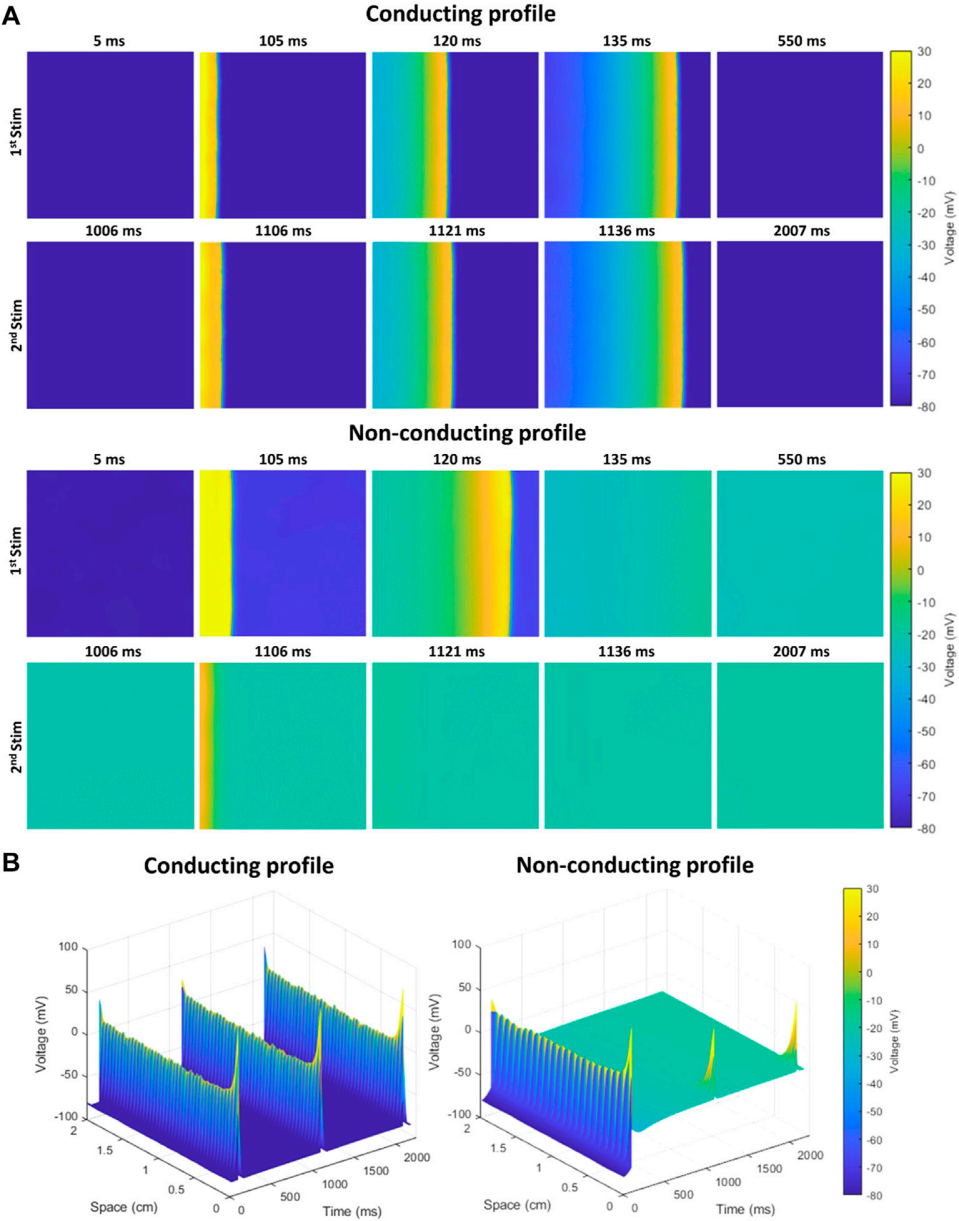
Example of conducting and non-conducting profiles both with 10% of fibrosis, in the absence of the effect of any antiarrhythmic drug. **(A)** Propagation of two stimuli in each profile across the simulated tissue (2 cm × 2 cm) from left to right, with no-flux boundary conditions. While the second impulse propagates in the first profile, it does not in the second profile as the simulated tissue is failing to repolarize to initial membrane potential. **(B)** Time-space plot along the propagation direction in both profiles, showing effective propagation in the first profile and the blockade in the second profile.

Several supervised models were trained to evaluate the algorithm that better described the behavior of the population including Quadratic Support Vector Machine (QSVM), Cubic Support Vector Machine (CSVM), decision trees (DT), K-Nearest Neighbors (KNN) with 10k-fold cross validation. The input of the algorithms corresponded to a combination of the variables from the population of models: g_Na_, I_NaK_, g_K1_, g_CaL_, g_Kur_, I_KCa_, diffusion (D), extracellular potassium concentration ([K]_o_) and extracellular sodium concentration ([Na]_o_), including the variation induced in the final conductance of the channel (G_i_) by the different antiarrhythmic drugs tested (amiodarone, dofetilide or sotalol), and the percentage of fibrosis (0, 5% or 10%). AI algorithms were trained to predict a binary outcome: conduction along the plane (labeled as 1) or absence of conduction (labeled as 0). An example of a conducting and non-conducting profile both with 10% fibrosis and in the absence of drugs are shown in Figure 1. Figure 1A shows how two consecutive impulses propagate in each profile across the simulated tissue and Figure 1B the time-space plot along the propagation direction. While at 10% fibrosis the combination of variables in the cardiomyocytes of the first profile allow to conduct both impulses, in the second profile, the different combination of variables produces a conduction block for the second stimulus. The cardiomyocyte variables in the conducting profile shown in Figure 1 correspond to the baseline reference Koivumaki Model with Skibsbye modifications (Skibsbye et al., 2016), and the variables in the non-conducting profile are: g_Na=+73.13%_, I_NaK=+76.95%_, g_K1=-38.37%_, g_CaL=+92.17%_, g_Kur=+77.18%_, I_KCa=-47.2%_, D_=-30.64%_, [K]_o=+94.93%_ and [Na]_o=+89.34%_ relative to the baseline cardiomyocyte model. Algorithms were trained using the variable parameters for the population of models while the output for prediction was if propagation was possible along the plane or not. Training testing ratio was set 80:20 as in previous experiments by the group (Sanchez de la Nava et al., 2021a).

The algorithms were evaluated by means of specificity, sensitivity, positive predictive value, negative predictive value and accuracy, as follows:

$$Sensitivity=\frac{Number\;of\;true\;positives}{Number\;of\;true\;positives+Number\;of\;false\;negatives}$$

$$Specificity=\frac{Number\;of\;true\;negatives\;}{Number\;of\;false\;positives+Number\;of\;true\;negatives}$$

$$Positive\;Predictive\;Value=\frac{Number\;of\;true\;positives\;}{Number\;of\;true\;positives+Number\;of\;false\;positives}$$

$$Negative\;Predictive\;Value=\frac{Number\;of\;true\;negatives\;}{Number\;of\;true\;negatives+Number\;of\;false\;negatives}$$

$$Accuracy=\frac{Number\;of\;correct\;predictions\;}{Total\;number\;of\;predictions}$$

### Variability on the simulated models: Validation of the protocol

In order to evaluate the potential applicability of this technology into different fields, and specially for translating the results into the clinic, we evaluated small variations on the population of models to quantify changes in propagation of the electrical impulse.

### Statistical analysis

The *t*-test was used to evaluate the statistical significance between continuous paired or unpaired variables. One-way ANOVA, Kruskal Wallis test calculator and Chi Square test were calculated to evaluate differences among the three studied groups (0% fibrosis, 5% fibrosis and 10% fibrosis) in continuous and binary variables, respectively. Statistical significance was considered for *p* <0.05 in all cases.

## Results

### Construction and calibration of the model population

**FIGURE 2:**
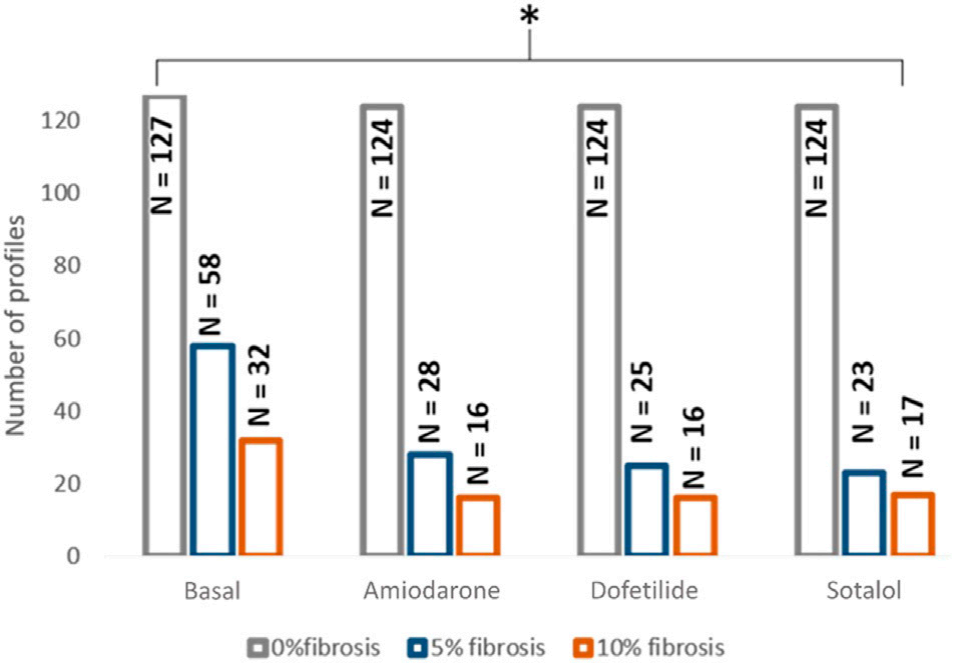
Distribution of the profiles in which propagation was observed depending on the fibrosis degree and the effect of the drug. * <0.05 for ANOVA statistical test.

From the complete population consisting on 127 different electrophysiological profiles that conducted the electrical impulse along the plane in 0% fibrosis conditions, 58 profiles conducted for the 5% condition and 32 for the 10% fibrosis (Figure 2). In addition, when the drugs were added, the number of conductive profiles diminished for all cases due to the antiarrhythmic effect of the compound (Figure 2). The distribution of the variables of the population of models that present propagation during simulations at three different fibrosis concentrations in basal conditions (no drugs) presented no significant differences and are shown in Supplementary Figure S1. All the subsequent results are presented for the profiles that did conduct the electrical impulse at 5% or at 10% fibrosis level.

**FIGURE 3:**
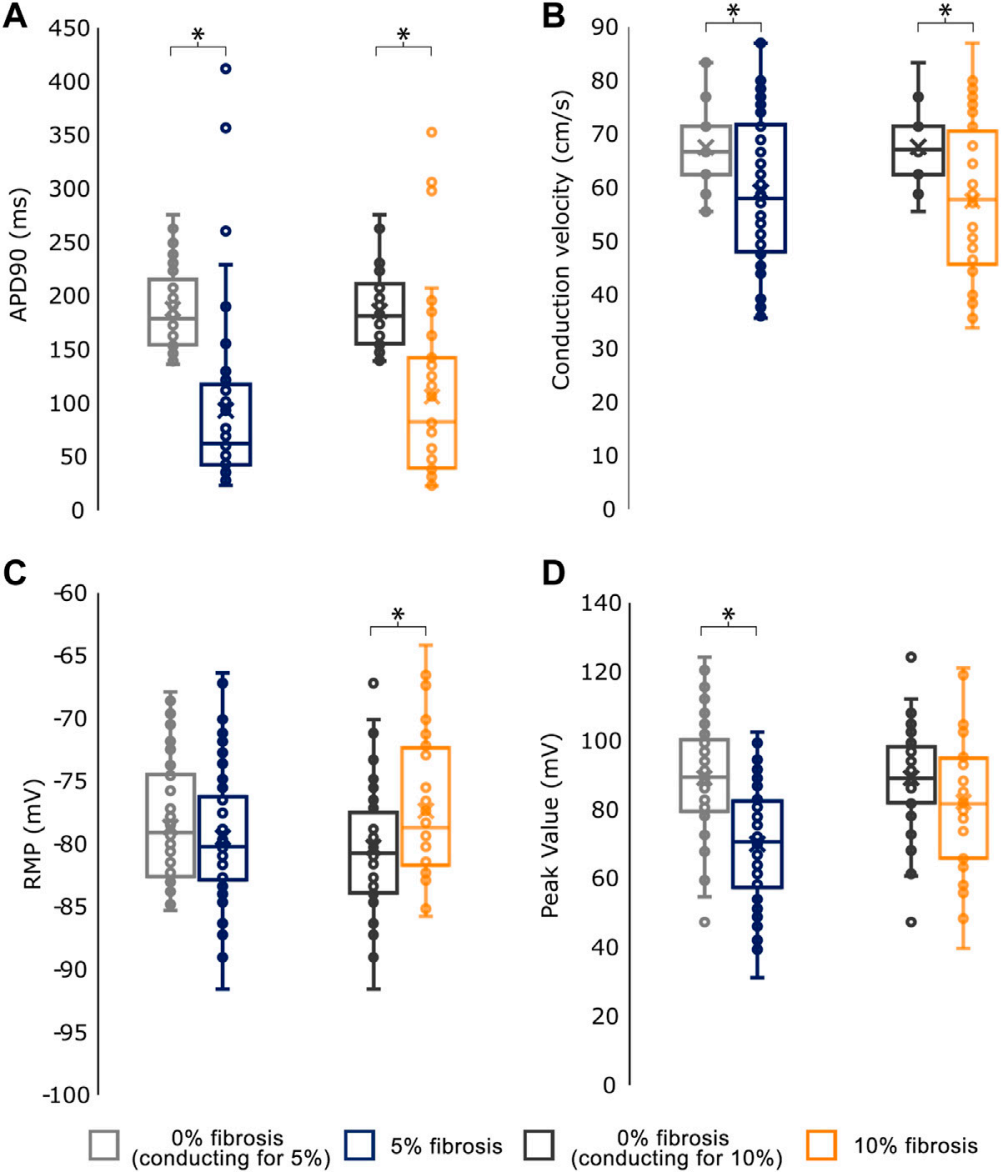
Mean values for the electrophysiological characterization of simulations conducting for the different fibroblast concentrations. **(A)** Action Potential Duration at 90% repolarization (APD90) **(B)** Conduction velocity **(C)** Resting Membrane Potential (RMP) and **(D)** Peak Transmembrane Value. As the number of profiles conducting in each group (0%, 5% and 10% fibrosis) differ, each group is compared against the basal (0% fibrosis) electrophysiological values of the same profiles.* <.05 for *t*-test statistical test.

The effects of fibrosis on the electrophysiological measurements in the simulations are shown in Figure 3. At the electrophysiological level, the presence of fibrosis produced a shortening of the APD90 (0% fibrosis: 187.77 ± 37.62 ms; 5% fibrosis: 93.29 ± 82.69 ms; 10% fibrosis: 106.37 ± 85.15 ms; *p*-value<0.001) on the simulated cardiomyocytes. Conduction velocity was significantly reduced for higher presence of fibrosis (0% fibrosis: 67.52 ± 7.3 cm/s; 5% fibrosis: 58.81 ± 14.04 cm/s; 10% fibrosis: 57.56 ± 14.78 cm/s; *p*-value<0.001). Both RMP (0% fibrosis: −78.63 ± 4.63 mV; 5% fibrosis: −79.46 ± 5.46 mV; 10% fibrosis: −77.35 ± 5.43 mV; *p*-value: >0.1) and peak membrane potential value in the cardiomyocytes (0% fibrosis: 89.16 ± 16.01 mV; 5% fibrosis: 70.06 ± 17.08 mV; 10% fibrosis: 82.21 ± 19.90 mV; *p*-value<0.001) presented a decrease for 5% fibrosis and an increase for 10% fibrosis planes (Figure 3).

### Drug implementation on the stable ionic profiles

All drugs were studied at two different levels on the conducting profiles at basal conditions: first, regarding the variations on the ionic conductances present at the population of models and secondly, based on the clinical biomarkers extracted from the simulations.

Figure 2 shows the distribution of the profiles conducting the electrical impulse for each scenario and Table 2 contains the proportion of profiles with conduction velocity modifications in the population with respect to the corresponding control population (0%, 5% or 10% fibrosis without drug). An example of a conducting profile and non-conducting profile for 10% fibrosis can be consulted in Figure 1. Interestingly, in the absence of fibrosis (0% fibrosis simulations), all the profiles presented a decrease in conduction velocity under the effect of all drugs, in accordance to what is described and expected for AADs. However, the simulations with fibrosis showed a different effect of some drugs: dofetilide and sotalol simulations exhibited part of the profiles in which the conduction velocity was increased (dofetilide: 28% of profiles; sotalol: 65.21%). Finally, when simulations were repeated with a higher percentage of fibrosis (i.e. 10% fibrosis) a dispersed effect on the conduction velocity was observed under the effect of all the tested drugs (increased conduction velocity of 31.25% for amiodarone profiles, 37.5% for dofetilide profiles and 43.75% for sotalol profiles).

**TABLE 2:** Change in conduction velocity for the studied antiarrhythmic drugs under the effect of fibrosis (number of profiles and percentage).

	0% Fibrosis		5% Fibrosis		10% Fibrosis	
	Increase in CV	Decrease in CV	Increase in CV	Decrease in CV	Increase in CV	Decrease in CV
Amiodarone	0	124 (100%)	0	28 (100%)	5 (31.25%)	11 (68.75%)
Dofetilide	0	124 (100%)	7 (28%)	13 (52%)	6 (37.5%)	3 (18.8%)
Sotalol	0	124 (100%)	15 (65.21%)	6 (26.08%)	7 (43.75%)	2 (12.5%)

In addition, the electrophysiological characterization of the simulations can be observed in Table 3, where the variation of four electrophysiological biomarkers (change in CV, APD90, RMP and peak value) induced by the different drugs is shown. Regarding the electrophysiological characterization, an average decrease or maintenance in CV was observed with respect to the corresponding control population, but the effect was more variable when fibrosis was present. The effect or drugs tended to reduce APD90 in most cases when measuring the variables in the cardiomyocytes except for the 5% fibrosis simulations. However, high variability in APD90 changes was observed among the different profiles. In the case of RMP, an average increase was observed on the 0% fibrosis under the effects of drugs, however, drugs tended to have the opposite effect and reduce RMP in the different profiles in the presence of fibrosis. The effect on RMP was highly variable for the different profiles, and in particular in the presence of dofetilide. Finally, the peak value tended to reduce in all cases except for the 10% fibrosis under the effect of dofetilide and sotalol.

**TABLE 3:** Change in conduction velocity for the studied antiarrhythmic drugs under the effect of fibrosis.

		ΔCV (cm/s)	*p*-value	ΔAPD90 (ms)	*p*-value	ΔRMP (mV)	*p*-value	ΔPeak value (mV)	*p*-value
Amiodarone	0% fibrosis	−3.59 ± 1.52	0.24	−49.42 ± 96.72	0.06	0.14 ± 0.43	<0.01	−4.76 ± 0.98	0.01
	5% fibrosis	−4.96 ± 2.15		−16.32 ± 93.05		−1.97 ± 3.15		−7.34 ± 3.61	
	10% fibrosis	0.62 ± 9.56		−28.79 ± 142.98		−5.39 ± 7.29		−1.97 ± 22.96	
Dofetilide	0% fibrosis	−13.43 ± 4.07	<0.01	−29.51 ± 71.67	0.92	1.78 ± 9.83	0.04	−18.85 ± 9.47	<0.01
	5% fibrosis	0.14 ± 1.87		5.73 ± 136.26		4.75 ± 27.58		−0.96 ± 3.83	
	10% fibrosis	0.05 ± 1.16		−18.92 ± 129.98		−1.94 ± 4.29		3.59 ± 11.31	
Sotalol	0% fibrosis	−0.023 ± 0.24	<0.01	−15.83 ± 43.76	0.06	0.18 ± 0.16	<0.01	−0.04 ± 0.16	<0.01
	5% fibrosis	0.30 ± 4.69		3.80 ± 135.56		−0.46 ± 3.34		−0.41 ± 3.12	
	10% fibrosis	0.22 ± 1.39		−40.28 ± 106.18		−1.93 ± 4.28		3.49 ± 11.49	

### *In Silico* models and artificial intelligence

**FIGURE 4:**
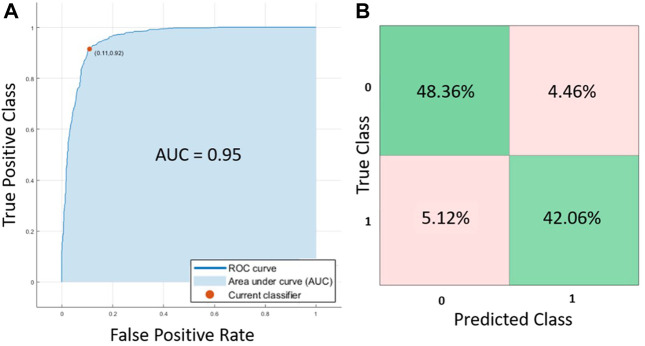
**(A)** ROC curves and **(B)** confusion matrix for the Cubic SVM trained algorithm.

Comparing the prediction accuracy of the different AI methods tested in this work, Cubic SVM showed the best performance for the tested dataset. As it can be observed from Table 4; Figure 4, the Area Under the Curve (AUC) was 0.95, the accuracy of the algorithm was 90.4% (C.I. 88.83%–91.85%) with a sensitivity of 90.43% (C.I. 88.20%–92.36%), specificity 90.41% (88.00%–92.48%), positive predictive value of 91.55% (C.I. 89.62%–93.15%) and negative predictive value of 89.15% (C.I. 86.92%–91.04%). All the other tested methods (QSVM, DT and KNN) showed an accuracy above 80% and an AUC larger than 0.89, but lower in comparison with CSVM.

**TABLE 4:** Evaluation metrics for the different AI algorithms trained including sensitivity, specificity and accuracy expressed in percentage and respective confidence interval in brackets, and Area Under the Curve (AUC).

	Sensitivity	Specificity	Accuracy	AUC
QSVM	84.67% [82.14%–86.96%]	93.11% [90.83%–94.97%]	88.12% [86.39%–89.71%]	0.94
CSVM	90.43% [88.20%–92.36%]	90.41% [88.00%–92.48%]	90.40% [88.83%–91.85%]	0.95
DT	79.85% [77.17%–82.35%]	92.93% [90.50%–94.90%]	84.71% [82.81%–86.48%]	0.89
KNN	78.65% [75.91%–81.22%]	90.05% [87.30%–92.38%]	82.94% [80.96%–84.80%]	0.92

The CSVM algorithm (Cristianini and Shawe-Taylor, 2000) includes an analysis to evaluate how important are the input features in outcome prediction. In particular, Sequential Minimal Optimization (Platt, 1998) that was implemented to solve the nonlinear problem during the algorithm training, enabled the identification and posterior optimization of the variables included in the final CSVM prediction algorithm. Once the problem has been solved, the kernel calculated for the support vector machines can reveal the relative importance of contributions during calibration by each of the input parameters. That is, their contribution to minimizing the error when predicting the outcome can be ranked based on quantifying their support vector magnitude. Utilizing such approach the CSVM algorithm revealed that the most important features for evaluating the block or conduction of the action potential in the tissue were in decreasing order: (i) the type of drug added, (ii) the values of the sodium currents (INak and gNa) and (iii) the values of the potassium currents (gK1 and gKur).

## Discussion

In this study, we present a new methodology to identify and predict the propagation of the electrical impulse in the presence of electrophysiological variability, the presence of different degrees of fibrosis and the effect of antiarrhythmic drugs. Several algorithms were trained and cubic SVM showed the best performance using the *in silico* data from the simulations. These simulations included: (i) electrophysiological variability using a population of models, (ii) substrate variability using different percentages of fibrosis, and (iii) drug effect variability, introduced by the implementation of the effect of three different antiarrhythmic drugs used in the clinical practice for AF treatment. The main result of the study showed that, depending on the percentage of fibrosis and the effect of the drug, the modifications of the conduction velocity of the substrate could lead to proarrhythmic scenarios in specific subgroups.

### Electrical conduction in 2D cardiac tissue: Effect of ionic profile and fibrosis presence

To our knowledge, this study is the first one that explores 2D tissue simulations combining a population of atrial cardiomyocytes with different percentages of diffused fibrosis. Previous studies have shown the effect of diffused fibrosis on the electrophysiological properties of the tissue, in line with the results presented in this publication (King et al., 2013; Palacio et al., 2021; Rios-Munoz et al., 2021). Our results are in agreement with studies that evaluated the effect of fibrosis in the electrophysiological characteristics of cardiac tissue showing a decrease in conduction velocity (Spencer et al., 2017).

### Drug effect on fibrotic tissue behaviour: Electrophysiological implications

Populations of models have been implemented in the cardiac scenario for the prediction of drug effect on cardiac tissue (Britton et al., 2013; Muszkiewicz et al., 2016; Sanchez de la Nava et al., 2021a; Peirlinck et al., 2021). Here, the 2D fibrotic tissue simulations were not only evaluated at basal conditions but also under the effect of different antiarrhythmic drugs, including amiodarone, dofetilide and sotalol. Overall, these antiarrhythmic drugs showed a total block in the majority of the profiles. In those in which electrical conduction was preserved, a decrease on conduction velocity was observed when simulations were performed in the 0% fibrosis tissue patch. This effect was preserved for amiodarone in the 5% fibrosis patch. Finally, 10% fibrosis simulations showed that part of the profiles increased CV while others showed a decrease in this variable.

This confirms our hypothesis that the expected effect of the drug can be altered due to the presence of fibrosis and that, for higher percentage of fibrosis, the effect is less predictable and more frequent to present unexpected effects. Furthermore, we observed that the antiarrhythmic drug that showed less variability of the effect among the different profiles was amiodarone, causing a decrease in the conduction velocity (CV) in most of the profiles with and without fibrosis. In contrast, the long-term use of amiodarone in porcine animal models with myocardial fibrosis showed no adverse effects (Zagorianou et al., 2016).

Reduced and more heterogeneous CV in the myocardium increases the probability of arrhythmia and can be caused by a single or combined effect of structural changes, alterations in electrical coupling in the tissue, or the effect of different drugs. In our study, the different profiles showed variability in the ionic current values and action potential morphology, leading to modifications of fibroblasts conduction velocity and arrhythmogenicity. Our results may help to further interpret the drug-induced modifications of CV in prior studies. Angiotensin converting enzyme inhibitors, angiotensin II type 1 receptor antagonists and pirfenidone have been shown to be effective in attenuating arrhythmogenic atrial remodeling, resulting in a marked reduction in atrial fibrosis, with reduced conduction heterogeneity and AF vulnerability (Li et al., 2001; Kumagai et al., 2003; Lee et al., 2006). On the other hand, the effects of drugs designed to enhance gap-junctional coupling on cardiac CV may depend on the presence of fibrotic changes. Rotigaptide showed that in the absence of fibroblasts, CV increases monotonically with gap junctional coupling (Lin et al., 2008). However, the presence of fibroblasts, resulted in a biphasic effect on CV, as showed in both experimental and computational studies (Miragoli et al., 2006; Zlochiver et al., 2008; Tveito et al., 2012). Additionally, the relative expression of Cx40 and Cx43 may either increase or decrease CV in experiments and humans (Bagwe et al., 2005; Dhillon et al., 2014). Finally, while acetylcholine induced APD shortening was able to induce an spiral wave AF episode in healthy canine hearts (Schuessler et al., 1992), shortening the APD using pinacidil induced AF was driven by intramural reentry anchored to atrial bundles insulated by fibrosis (Hansen et al., 2015). Therefore, we expect that using mathematical models such as used here may help to improve the understanding of the complex relationship of cardiac electrical substrate, electrical conduction and drugs effects on propagation and arrhythmogenicity.

### Artificial intelligence for the evaluation of tissue conduction

*In silico* simulations allow to produce a significant number of scenarios that is suitable for the application of AI algorithms that enable to better analyze and extract patterns. The use of this technology has increased exponentially in the last years with the aim of better predicting and identifying new biomarkers.

In this case, these algorithms were implemented with the main objective of identifying the capabilities of the tissue to conduct the electrical signal. Lack of conduction of a small patch in the heart has been proved to cause arrhythmia, described as anatomical reentry, where the anatomical pathway is fixed (Arenal et al., 2012). Among the algorithms explored in this study, all showed an accuracy above 80%. However, Cubic SVM showed the best performance compared to QSVM, DT and KNN, with an accuracy of 90.4% and an AUC of 0.95. This high accuracy highlights the potential of these techniques as collaborative and predictive tools in the clinic. Using this methodology in more patient-specific complex scenarios is more likely to predict the potential benefit of an antiarrhythmic drug on a specific patient by avoiding the occurrence of conduction blocks. For example, after further validation, using patient specific 3D geometry and fibrosis distribution obtained by MRI and combining it with this *in silico* model, the CSVM algorithm could be applied to help in the decision of which antiarrhythmic drug may be more appropriate.

### Limitations

The main limitations of this study include the high computational cost associated to obtain a broad number of profiles. In the same line, simulations were performed in 2D planes to reduce the overall computational cost, but the study will benefit to include more complex structures in the model such as the pulmonary veins, different CV areas (Sánchez et al., 2019) or a 3D configuration.

Although we considered the variability of the cardiomyocyte population, new approaches should explore the possibility of developing a population for human fibroblasts to be included in the model, as for this study variability was only introduced in the cardiomyocyte model.

Finally, regarding the diffused fibrosis that has been studied, the percentage of fibroblasts was varied from 0% to 5% and 10%, but the distribution of the fibroblast was determined randomly for each percentage and kept constant for all the profiles. Running the different profiles with the same percentage of fibrosis, but different distributions should be further tested in order to corroborate the obtained results.

### Clinical implications

The identification of specific scenarios and combination of variables that present proarrhythmic effects can be of great importance in the understanding and development of new tools for future pharmacological treatments in the concepts of personalized medicine and optimizing treatment efficacy.

## Conclusion

Cardiac fibrosis can alter the expected behavior of antiarrhythmic drugs in a minority of the population and data analysis using artificial intelligence can reveal the profiles that will respond differently.

## Data Availability

The code for the cardiomyocyte model, the fibroblast model, and the population of models are provided in the Supplementary Figure S1 (Supplementary Data S1, S2). The rest of the raw data supporting the conclusion of this article will be made available by the authors, without undue reservation.
