# Supplementary material for: Artificial intelligence analysis of the impact of fibrosis in arrhythmogenesis and drug response
Source: Front Physiol. 2022 Oct 12;13:1025430. doi: 10.3389/fphys.2022.1025430 (PMC9596790; doi:10.3389/fphys.2022.1025430)
Supplement: Supplementary file 1 [file Table1.docx]

**Supplementary Table S1**: Parameters used in the baseline cardiomyocyte model after AF remodeling (first row) and the variations introduced for generating the different profiles in the population of models. Each row corresponds to a different profile in the population and to the corresponding values for each parameter, shown in the variation percentage with respect to the baseline cardiomyocyte model.

| **g_Na_** | **I_NaK max_** | **g_K1_** | **g_CaL_** | **g_Kur_** | **g_KCa_** | **Diffusion (D)** | **[K]_o_** | **[Na]_o_** |
| --- | --- | --- | --- | --- | --- | --- | --- | --- |
| **524.8** | **70.83** | **4.70** | **2.93** | **1.395** | **1.8** | **100%** | **5.4 mM** | **130 mM** |
| -32.19% | +71.41% | +32.89% | +46.79% | -0.79% | -0.04% | +0.48% | -48.46% | +40.23% |
| +57.25% | -27.60% | -21.13% | -15.02% | +96.04% | +14.19% | +2.71% | +24.39% | -0.48% |
| +73.13% | +76.95% | -38.37% | +92.17% | +77.18% | -47.20% | -30.64% | +94.93% | +89.34% |
| +59.56% | -11.43% | +45.63% | +17.38% | -10.62% | +98.20% | -20.86% | +69.11% | +80.07% |
| -17.98% | +50.85% | -40.74% | +95.42% | -49.17% | +85.44% | +64.50% | +89.80% | -42.42% |
| +40.34% | -4.38% | -34.20% | -29.20% | +36.03% | -19.27% | +11.33% | +97.89% | -15.88% |
| +47.40% | +11.15% | +20.15% | +40.23% | +22.04% | +69.47% | -17.07% | -16.43% | -42.78% |
| -5.97% | +99.31% | +0.68% | +76.35% | +83.44% | +1.69% | -27.97% | +53.87% | +50.91% |
| +46.21% | +88.06% | -33.51% | +80.44% | -12.84% | -39.95% | +5.16% | +17.89% | +87.85% |
| +10.90% | -32.44% | +4.83% | +34.36% | -19.48% | +68.18% | +27.00% | +52.68% | +97.78% |
| +76.19% | +17.21% | +8.70% | +95.14% | -18.16% | -42.58% | +73.10% | +33.65% | +3.28% |
| +61.91% | +76.08% | -45.62% | -15.37% | -47.80% | +94.84% | +45.07% | +45.17% | +83.22% |
| -6.66% | +18.06% | -16.10% | +83.15% | +89.80% | -22.20% | +32.31% | +59.73% | -1.34% |
| +50.56% | +87.44% | +16.75% | +42.43% | -38.26% | +28.40% | +47.22% | +31.12% | +41.67% |
| +2.03% | +33.93% | +30.74% | -32.06% | -3.84% | -15.78% | -11.01% | -14.38% | -36.74% |
| +35.19% | -7.00% | +26.35% | +83.58% | -32.40% | -41.72% | +11.67% | +10.81% | +93.05% |
| -10.82% | +1.18% | +48.07% | +46.00% | +94.41% | +30.63% | -3.94% | +45.93% | +34.07% |
| +98.97% | +83.09% | +35.00% | +93.07% | -16.18% | -6.28% | -0.43% | -17.67% | +92.80% |
| -13.27% | -15.10% | -11.84% | +65.09% | +69.38% | +13.54% | +12.63% | +82.75% | +94.59% |
| +46.34% | -3.08% | +14.21% | -21.29% | -40.93% | +57.60% | +3.17% | +56.30% | -37.90% |
| +49.02% | -4.66% | +21.82% | +58.38% | +44.27% | +2.54% | +1.04% | +78.57% | +96.92% |
| -38.26% | -47.73% | +13.43% | -48.51% | +33.40% | +61.80% | -23.33% | +13.28% | +45.16% |
| +72.72% | +88.53% | -40.45% | +13.76% | +63.44% | +14.48% | +4.59% | -14.91% | +76.26% |
| +42.32% | +43.77% | -16.89% | +55.65% | -33.06% | +64.94% | +34.47% | +67.33% | +1.26% |
| +83.87% | +71.65% | -23.42% | +69.54% | +27.61% | -45.48% | -47.34% | +10.41% | +66.90% |
| +20.38% | +74.42% | +22.39% | +61.48% | -45.58% | +94.08% | +24.79% | +36.98% | +40.77% |
| +38.94% | -8.06% | +70.14% | +50.72% | +23.44% | +54.84% | -2.20% | +20.61% | +81.68% |
| -9.93% | +91.53% | -24.95% | +77.59% | +3.81% | +74.38% | +9.66% | +77.11% | +1.44% |
| +80.50% | +58.50% | -22.26% | +22.32% | +47.40% | +25.82% | -15.98% | +24.42% | +12.11% |
| +17.98% | +57.26% | -44.64% | -4.34% | +99.31% | +69.74% | -24.18% | +50.08% | +60.25% |
| +35.22% | +37.33% | +16.90% | +23.30% | -9.37% | +21.44% | -17.46% | +80.36% | +59.06% |
| -26.55% | +13.51% | +69.06% | -0.08% | -7.35% | -25.88% | -0.65% | +57.78% | -9.58% |
| -38.80% | -28.17% | +60.01% | +51.11% | -17.77% | +56.89% | -6.94% | -40.83% | +86.22% |
| +89.42% | -44.59% | +69.60% | +66.36% | -9.90% | +77.99% | -5.43% | +37.12% | +12.50% |
| -49.71% | +81.75% | +28.39% | -34.47% | -4.76% | +73.97% | -5.98% | +76.51% | +63.51% |
| +28.20% | +0.05% | -32.91% | +15.24% | +80.81% | -0.39% | -18.87% | +44.06% | +0.69% |
| +10.84% | +50.69% | -39.28% | -25.25% | +50.34% | -22.69% | -16.62% | +88.92% | +76.93% |
| +42.96% | +66.62% | -45.33% | +59.46% | +79.77% | +40.81% | +28.15% | +69.07% | +42.67% |
| -15.92% | -34.13% | +42.65% | -18.27% | +58.84% | -25.08% | -9.98% | -35.17% | +89.69% |
| +25.21% | +79.65% | -4.63% | +37.25% | +21.93% | -4.82% | -13.41% | +5.89% | +75.67% |
| +92.52% | -44.06% | +63.86% | +71.29% | +41.09% | +40.32% | -14.09% | -35.34% | +70.75% |
| -16.20% | -38.56% | +29.97% | +83.25% | +37.59% | -34.29% | +8.95% | +57.68% | -26.88% |
| +41.14% | +74.82% | -8.31% | +90.99% | +41.51% | -11.12% | +42.80% | -42.65% | +75.74% |
| +33.11% | -26.27% | -11.46% | +61.79% | +65.72% | +90.09% | +13.90% | +47.95% | -23.18% |
| -32.83% | -39.94% | +44.40% | +64.72% | +80.05% | -43.12% | -19.80% | +18.57% | +24.90% |
| +66.29% | +16.73% | -4.77% | +42.32% | +3.59% | +7.56% | +40.28% | -41.99% | -25.37% |
| +14.58% | +82.78% | -35.44% | -44.39% | +60.13% | -13.26% | -29.33% | +67.15% | -7.13% |
| +84.40% | +77.58% | -31.80% | +9.02% | -36.51% | +34.57% | -0.06% | -33.15% | +60.00% |
| +47.57% | +87.01% | -23.77% | +73.02% | -48.88% | +97.86% | -26.86% | +3.83% | -0.07% |
| +3.34% | +66.32% | +11.52% | +23.84% | +10.43% | +51.63% | +9.82% | +73.52% | -38.63% |
| +13.76% | +34.56% | -46.11% | -7.08% | +10.96% | +49.50% | +1.31% | +62.68% | +65.63% |
| +86.65% | -34.54% | -1.55% | +89.78% | +63.34% | -25.16% | +54.47% | +73.85% | +26.27% |
| +18.65% | +63.22% | -12.75% | +96.95% | -23.04% | +85.95% | -9.18% | -6.54% | -29.32% |
| +9.97% | +59.80% | +1.67% | -12.22% | -27.01% | +2.80% | -21.80% | -41.60% | +35.51% |
| +55.80% | -31.03% | +14.83% | -4.91% | +47.93% | -30.84% | -24.62% | +71.70% | -36.88% |
| +49.68% | +69.09% | -26.19% | +17.72% | -6.64% | -35.82% | +35.52% | -39.98% | +81.10% |
| +57.53% | +31.68% | +40.68% | +99.23% | -49.66% | +85.61% | +57.28% | +1.23% | -3.44% |
| +6.81% | +35.35% | -25.92% | +14.59% | +1.69% | -8.29% | -17.64% | -10.45% | -31.54% |
| +48.73% | +4.41% | +51.86% | +75.70% | -34.48% | -8.65% | -15.13% | +8.09% | +72.35% |
| +67.70% | +5.03% | -19.39% | +17.12% | +29.83% | -43.91% | -32.85% | +88.15% | +23.33% |
| -14.96% | -28.90% | +8.27% | -46.39% | -5.63% | +31.77% | -33.38% | +46.74% | +79.39% |
| +82.46% | -0.45% | -45.90% | -42.77% | +55.32% | +41.34% | -22.54% | -20.84% | +17.18% |
| +61.63% | +94.85% | +17.42% | -1.69% | -43.16% | -29.08% | -9.29% | +60.77% | -11.41% |
| +71.79% | +46.75% | -18.55% | +13.95% | +10.21% | +94.93% | +3.72% | -20.45% | -45.63% |
| +14.06% | +44.93% | +21.11% | +93.71% | +28.76% | -36.59% | +7.61% | +48.18% | -14.70% |
| -32.93% | +13.27% | -9.21% | +24.43% | +44.80% | +96.66% | -12.38% | +82.92% | +48.87% |
| +27.06% | +80.38% | +81.46% | +31.25% | -40.34% | -29.69% | -9.79% | +47.22% | -3.83% |
| +68.12% | -17.14% | +5.31% | +68.30% | +94.88% | -7.97% | -31.39% | +32.07% | +85.70% |
| -3.59% | +98.33% | +25.16% | +5.82% | -8.86% | +83.49% | -7.49% | +90.93% | +40.99% |
| +92.23% | -22.04% | -44.55% | +12.26% | +2.97% | +86.99% | +6.37% | -18.65% | -24.17% |
| +44.00% | +95.64% | -43.60% | -9.45% | +12.73% | -26.28% | +4.08% | +34.09% | -40.43% |
| +2.87% | +34.23% | +9.53% | +18.47% | -13.42% | +47.47% | -26.32% | +6.94% | -39.98% |
| +48.66% | +70.85% | -49.95% | +36.38% | +93.93% | -24.14% | -25.61% | -28.36% | -49.51% |
| -5.51% | +91.87% | +18.78% | +73.87% | -23.51% | +5.48% | -3.25% | +18.80% | +70.91% |
| +90.79% | -39.04% | +17.94% | +81.81% | -24.71% | +69.12% | +29.07% | +2.22% | -21.50% |
| -30.97% | -32.72% | +52.17% | -37.85% | +13.42% | +61.30% | -12.13% | +23.35% | +6.59% |
| +38.77% | +36.56% | -26.64% | +11.84% | -1.11% | +8.76% | -11.61% | +85.56% | -37.53% |
| -7.43% | +36.26% | -34.68% | +91.56% | -11.53% | +50.91% | +10.01% | -45.91% | -2.48% |
| +78.34% | -2.68% | +15.85% | +93.47% | -22.39% | +96.03% | +34.27% | -46.47% | +91.87% |
| +37.36% | -13.09% | +38.10% | +70.22% | -36.90% | -5.47% | +21.62% | +84.44% | +74.33% |
| +79.49% | +14.56% | -19.84% | -26.42% | -40.44% | +60.03% | -8.04% | +53.73% | +39.35% |
| -27.25% | +12.73% | +18.29% | +72.69% | -49.85% | +80.89% | +14.73% | +14.56% | +20.17% |
| +89.13% | -21.57% | -43.80% | -5.20% | +54.59% | -45.09% | +28.39% | +62.14% | +46.94% |
| +8.73% | +2.38% | -9.02% | +0.68% | -22.43% | +32.49% | +12.20% | +71.91% | -25.07% |
| +59.33% | -14.40% | -25.47% | +26.52% | -27.83% | +56.80% | -14.76% | +42.64% | +50.13% |
| +39.17% | +46.30% | -14.55% | +28.46% | +29.09% | +20.37% | +0.15% | -6.18% | -47.41% |
| +87.12% | -33.38% | -0.87% | +56.90% | +46.83% | +77.74% | -0.85% | +70.73% | +67.76% |
| -9.18% | +47.17% | -6.50% | +67.94% | +31.87% | +35.32% | -10.14% | -22.04% | +93.61% |
| -11.35% | -7.33% | +23.76% | +1.64% | +19.87% | +19.64% | +5.87% | +53.06% | +22.32% |
| +60.11% | -35.77% | +6.93% | +7.16% | -44.51% | +48.12% | +26.33% | -28.89% | +36.07% |
| -2.69% | -2.56% | +10.93% | -37.31% | +41.87% | -0.71% | -32.05% | -11.18% | +37.64% |
| +36.39% | -1.29% | +3.35% | +21.95% | -23.98% | +79.64% | +53.43% | -4.18% | -7.73% |
| +80.97% | +29.80% | +25.46% | +92.28% | -16.93% | +28.73% | +24.30% | +54.33% | +25.12% |
| +30.58% | -48.21% | +38.58% | +71.51% | -34.35% | +73.24% | -21.82% | +89.39% | -33.03% |
| +62.55% | -23.00% | -13.79% | +30.92% | +45.77% | +68.59% | +0.70% | -32.31% | +77.92% |
| +11.59% | +9.21% | -3.44% | -16.73% | +72.04% | +42.13% | -23.65% | -25.58% | +69.89% |
| -17.31% | -31.44% | +72.05% | +26.97% | +25.99% | -20.51% | -6.56% | +78.26% | +94.99% |
| +84.72% | +62.40% | -35.22% | +16.08% | +17.00% | -39.29% | -40.63% | +9.42% | +61.64% |
| +53.86% | +60.67% | -48.29% | -33.63% | +40.81% | -11.45% | -2.32% | -21.72% | +27.09% |
| -17.30% | -30.20% | +45.26% | +42.08% | +48.13% | -43.59% | +21.75% | -7.40% | -33.22% |
| +99.38% | -25.15% | +59.38% | +78.30% | -44.27% | +31.32% | +22.65% | -40.64% | -14.28% |
| +11.44% | +9.66% | +89.60% | +52.52% | -30.90% | -33.01% | -2.66% | +66.01% | +27.36% |
| +65.50% | -20.62% | +35.34% | +48.52% | -33.73% | -44.28% | +15.66% | +91.30% | +71.35% |
| +85.17% | +56.11% | -35.94% | +71.87% | -30.24% | +17.86% | -18.20% | +91.03% | -30.85% |
| +98.33% | +47.30% | -42.05% | +60.58% | -30.50% | +49.01% | +69.31% | +98.81% | -14.39% |
| +55.52% | -4.95% | -8.76% | +87.85% | +31.37% | +29.59% | +44.44% | +64.74% | -19.52% |
| -21.79% | +48.30% | -4.22% | +92.57% | +43.51% | +38.19% | +12.76% | -29.46% | +61.44% |
| -43.97% | +97.59% | +12.28% | -5.80% | +6.26% | -48.61% | -4.52% | +56.07% | -5.40% |
| +38.48% | -12.32% | -21.28% | -26.29% | +59.37% | +40.93% | -7.14% | -0.44% | +71.80% |
| +28.38% | +2.71% | +25.79% | +6.64% | -20.99% | -46.59% | +41.08% | +78.85% | -5.67% |
| +28.77% | -18.00% | -3.84% | +27.70% | +74.29% | -10.89% | +22.23% | +61.36% | -33.94% |
| +70.71% | +3.83% | -24.01% | -35.51% | +28.37% | +84.90% | -33.13% | +41.06% | +49.80% |
| -37.46% | +77.26% | +96.84% | +94.85% | -11.96% | +70.89% | -1.64% | -18.49% | +15.20% |
| +79.65% | +7.30% | +1.34% | +79.86% | +0.15% | +42.68% | +43.34% | +86.69% | -11.27% |
| -16.90% | -16.17% | -28.18% | +4.69% | +40.23% | +27.91% | -3.57% | +45.03% | +73.66% |
| +41.45% | +49.62% | +36.96% | +60.03% | -1.62% | +35.81% | -1.11% | +93.62% | -43.25% |
| +67.00% | +52.07% | +41.15% | +45.51% | +41.37% | +97.18% | -12.98% | -21.01% | -34.40% |
| +0.39% | +28.94% | -0.49% | -11.31% | -0.13% | +17.40% | +21.11% | +27.83% | +73.26% |
| +49.34% | +75.72% | -28.53% | +23.71% | +73.15% | +3.78% | +24.61% | +97.27% | -14.92% |
| +8.48% | +0.37% | +53.41% | +44.63% | +92.23% | +39.59% | -20.18% | +90.42% | +3.73% |
| -19.55% | -23.96% | +23.35% | -1.34% | +51.26% | -16.90% | -25.99% | +34.46% | +33.48% |
| +84.47% | +17.15% | -32.90% | +22.85% | -35.27% | +75.64% | -8.36% | -44.70% | -23.63% |
| -16.56% | +48.69% | -1.21% | +32.58% | +76.53% | -16.20% | +13.05% | +38.94% | +69.39% |
| -10.13% | +25.53% | -30.39% | +37.85% | +99.84% | -32.81% | +4.83% | +25.59% | -12.06% |
| +67.21% | -10.43% | +2.28% | +70.54% | +55.61% | -40.94% | -12.57% | +33.02% | +8.42% |
| +5.12% | +87.12% | -10.17% | -4.59% | +20.77% | +34.79% | -24.49% | +43.41% | +22.81% |
