# Supplementary material for: Artificial intelligence analysis of the impact of fibrosis in arrhythmogenesis and drug response
Source: Front Physiol. 2022 Oct 12;13:1025430. doi: 10.3389/fphys.2022.1025430 (PMC9596790; doi:10.3389/fphys.2022.1025430)
Supplement: Supplementary file 2 [file Table2.docx]

**Supplementary Table S2**: Parameters used in the active fibroblast model.

| **g_bNa_** | **I_NaK max_** | **g_Kv_** | **g_Ki_** |
| --- | --- | --- | --- |
| **0.0095 nS/pF** | **1.644 pA/pF** | **0.25 nS/pF** | **0.4822 nS/pF** |
