# Supplementary material for: Artificial intelligence analysis of the impact of fibrosis in arrhythmogenesis and drug response
Source: Front Physiol. 2022 Oct 12;13:1025430. doi: 10.3389/fphys.2022.1025430 (PMC9596790; doi:10.3389/fphys.2022.1025430)
Supplement: Supplementary file 3 [file Table3.docx]

**Supplementary Table 3**: Human Atrial Action Potential biomarker ranges in AF (Simon et al., 2017).

|  | **Minimum Value** | **Maximum Value** |
| --- | --- | --- |
| **APD90 (ms)** | 140 | 330 |
| **APD50 (ms)** | 30 | 180 |
| **APD20 (ms)** | 1 | 75 |
| **APA (mV)** | 80 | 130 |
| **RMP (mV)** | -85 | -65 |
| **V20 (mV)** | -30 | 20 |
