# Supplementary material for: Artificial intelligence analysis of the impact of fibrosis in arrhythmogenesis and drug response
Source: Front Physiol. 2022 Oct 12;13:1025430. doi: 10.3389/fphys.2022.1025430 (PMC9596790; doi:10.3389/fphys.2022.1025430)
Supplement: Supplementary file 4 [file DataSheet2.DOCX]

__device__ void currents_rl_expl(state_variables &rN, state_variables &Deriv, float tim,int cell, int modelo)

{

float Ena,Ek,Eca,Der_v,Stim,Vshar,Vvecinos;

float a,pi,k1,k2,k3,b;

float V1,V2,V3,V4,VSR1,VSR2,VSR3,VSR4;

float JsercaSR1,JsercaSR2,JsercaSR3,JsercaSRss,J1serca,J2serca,J3serca,Jssserca;

float RyRsrCa1,RyRsrCa2,RyRsrCa3,RyRsrCass,Jrel1,Jrel2,Jrel3,Jrelss,Jsrleak1,Jsrleak2,Jsrleak3,Jsrleakss;

float Jj_nj,JCa1,JCa2,JCa3,JCa4,JCass,JSRCa1,JSRCa2,JSRCa3,JSRCa4,JNa;

float betass,betai1,betai2,betai3,betai4,gammai1,gammai2,gammai3,gammai4,betaSR1,betaSR2,betaSR3,betaSR4,betaNass;

int cond,ind;

float cte, rem,val,cKo,cNao,cD;

cte=0.00001f*(conducg2.cond_vecino8[modelo]);//Difussion variability

//S1 stimuli

a=floor((tim-S1_beging)/S1_periodg);

cond=(tipo_celg.Stim1[cell]==1)&&(tim<S1_beging+a*S1_periodg+S1_total_timeg)&&(a>=-0.01f);

Stim=cond*S1_ampg;

//S2 stimuli

b=floor((tim-S2_beging)/S2_periodg);

cond=(tipo_celg.Stim2[cell]==1)&&(tim<S2_beging+b*S2_periodg+S2_total_timeg)&&(b>=-0.01f);

Stim+=cond*S2_ampg;

Der_v=0.0f;

Vshar=rN.V[cell];

// Neighbour cells

Vvecinos=0.0f;

ind=vecinosg.vecino0[cell];

if ((ind>-1) && (tipo_celg.tipo[cell]==1)){

Vvecinos+=cte*conducg.cond_vecino0[cell]*(Vshar-rN.V[ind]); //(Estas lecturas , rN.V[ind] no son coalesced

}else if (ind>-1){

Vvecinos+=0.25f*cte*conducg.cond_vecino0[cell]*(Vshar-rN.V[ind]);

}

//

ind=vecinosg.vecino1[cell];

if ((ind>-1) && (tipo_celg.tipo[cell]==1)){

Vvecinos+=cte*conducg.cond_vecino1[cell]*(Vshar-rN.V[ind]); //(Estas lecturas , rN.V[ind] no son coalesced

}else if (ind>-1){

Vvecinos+=0.25f*cte*conducg.cond_vecino1[cell]*(Vshar-rN.V[ind]);

}

//

ind=vecinosg.vecino2[cell];

if ((ind>-1) && (tipo_celg.tipo[cell]==1)){

Vvecinos+=cte*conducg.cond_vecino2[cell]*(Vshar-rN.V[ind]); //(Estas lecturas , rN.V[ind] no son coalesced

}else if (ind>-1){

Vvecinos+=0.25f*cte*conducg.cond_vecino2[cell]*(Vshar-rN.V[ind]);

}

//

ind=vecinosg.vecino3[cell];

if ((ind>-1) && (tipo_celg.tipo[cell]==1)){

Vvecinos+=cte*conducg.cond_vecino3[cell]*(Vshar-rN.V[ind]); //(Estas lecturas , rN.V[ind] no son coalesced

}else if (ind>-1){

Vvecinos+=0.25f*cte*conducg.cond_vecino3[cell]*(Vshar-rN.V[ind]);

}

//

ind=vecinosg.vecino4[cell];

if ((ind>-1) && (tipo_celg.tipo[cell]==1)){

Vvecinos+=cte*conducg.cond_vecino4[cell]*(Vshar-rN.V[ind]); //(Estas lecturas , rN.V[ind] no son coalesced

}else if (ind>-1){

Vvecinos+=0.25f*cte*conducg.cond_vecino4[cell]*(Vshar-rN.V[ind]);

}

//

ind=vecinosg.vecino5[cell];

if ((ind>-1) && (tipo_celg.tipo[cell]==1)){

Vvecinos+=cte*conducg.cond_vecino5[cell]*(Vshar-rN.V[ind]); //(Estas lecturas , rN.V[ind] no son coalesced

}else if (ind>-1){

Vvecinos+=0.25f*cte*conducg.cond_vecino5[cell]*(Vshar-rN.V[ind]);

}

//

ind=vecinosg.vecino6[cell];

if ((ind>-1) && (tipo_celg.tipo[cell]==1)){

Vvecinos+=cte*conducg.cond_vecino6[cell]*(Vshar-rN.V[ind]); //(Estas lecturas , rN.V[ind] no son coalesced

}else if (ind>-1){

Vvecinos+=0.25f*cte*conducg.cond_vecino6[cell]*(Vshar-rN.V[ind]);

}

//

ind=vecinosg.vecino7[cell];

if ((ind>-1) && (tipo_celg.tipo[cell]==1)){

Vvecinos+=cte*conducg.cond_vecino7[cell]*(Vshar-rN.V[ind]); //(Estas lecturas , rN.V[ind] no son coalesced

}else if (ind>-1){

Vvecinos+=0.25f*cte*conducg.cond_vecino7[cell]*(Vshar-rN.V[ind]);

}

//

ind=vecinosg.vecino8[cell];

if ((ind>-1) && (tipo_celg.tipo[cell]==1)){

Vvecinos+=cte*conducg.cond_vecino8[cell]*(Vshar-rN.V[ind]); //(Estas lecturas , rN.V[ind] no son coalesced

}else if (ind>-1){

Vvecinos+=0.25f*cte*conducg.cond_vecino8[cell]*(Vshar-rN.V[ind]);

}

//

ind=vecinosg.vecino9[cell];

if ((ind>-1) && (tipo_celg.tipo[cell]==1)){

Vvecinos+=cte*conducg.cond_vecino9[cell]*(Vshar-rN.V[ind]); //(Estas lecturas , rN.V[ind] no son coalesced

}else if (ind>-1){

Vvecinos+=0.25f*cte*conducg.cond_vecino9[cell]*(Vshar-rN.V[ind]);

}

//

ind=vecinosg.vecino10[cell];

if ((ind>-1) && (tipo_celg.tipo[cell]==1)){

Vvecinos+=cte*conducg.cond_vecino10[cell]*(Vshar-rN.V[ind]); //(Estas lecturas , rN.V[ind] no son coalesced

}else if (ind>-1){

Vvecinos+=0.25f*cte*conducg.cond_vecino10[cell]*(Vshar-rN.V[ind]);

}

//

ind=vecinosg.vecino11[cell];

if ((ind>-1) && (tipo_celg.tipo[cell]==1)){

Vvecinos+=cte*conducg.cond_vecino11[cell]*(Vshar-rN.V[ind]); //(Estas lecturas , rN.V[ind] no son coalesced

}else if (ind>-1){

Vvecinos+=0.25f*cte*conducg.cond_vecino11[cell]*(Vshar-rN.V[ind]);

}

//

ind=vecinosg.vecino12[cell];

if ((ind>-1) && (tipo_celg.tipo[cell]==1)){

Vvecinos+=cte*conducg.cond_vecino12[cell]*(Vshar-rN.V[ind]); //(Estas lecturas , rN.V[ind] no son coalesced

}else if (ind>-1){

Vvecinos+=0.25f*cte*conducg.cond_vecino12[cell]*(Vshar-rN.V[ind]);

}

//

ind=vecinosg.vecino13[cell];

if ((ind>-1) && (tipo_celg.tipo[cell]==1)){

Vvecinos+=cte*conducg.cond_vecino13[cell]*(Vshar-rN.V[ind]); //(Estas lecturas , rN.V[ind] no son coalesced

}else if (ind>-1){

Vvecinos+=0.25f*cte*conducg.cond_vecino13[cell]*(Vshar-rN.V[ind]);

}

//

ind=vecinosg.vecino14[cell];

if ((ind>-1) && (tipo_celg.tipo[cell]==1)){

Vvecinos+=cte*conducg.cond_vecino14[cell]*(Vshar-rN.V[ind]); //(Estas lecturas , rN.V[ind] no son coalesced

}else if (ind>-1){

Vvecinos+=0.25f*cte*conducg.cond_vecino14[cell]*(Vshar-rN.V[ind]);

}

//

Der_v=Vvecinos;

//CM equations

if (tipo_celg.tipo[cell]==1) {

cKo=0.00001f*(conducg2.cond_vecino6[modelo]);

cNao=0.00001f*(conducg2.cond_vecino7[modelo]);

//Equillibrium potentials

Ena = RTFg*log((cNao*Naog)/rN.Nass[cell]);

Ek = RTFg*log((cKo*Kog)/rN.Ki[cell]);

Eca = RTFg*0.5f*log(Caog/rN.Cass[cell]);

Deriv.Nass[cell] = 0.0f;

Deriv.Ki[cell] = 0.0f;

Deriv.Cass[cell] = 0.0f;

//Sodium current

val=0.00001f*conducg2.cond_vecino0[modelo]; //Population of models

a = val*gnag*powf(rN.m[cell],3.0f)*(rN.h1[cell])*(rN.h2[cell])*(Vshar - Ena);

Der_v += a;

Deriv.Nass[cell]-= a;

//INaL current

a = glnag*powf(rN.m[cell],3.0f) * rN.fl1[cell]*(Vshar - Ena);

Der_v += a;

Deriv.Nass[cell] -= a;

// Ik1 current

val=0.00001f*((conducg2.cond_vecino2[modelo])); //Population of models

a = val*gk1g*powf(cKo*Kog,0.4457f)*(Vshar-Ek)/(1.0f+exp(1.5f*(Vshar-Ek+3.6f)/RTFg));//Ik1

Der_v += a;

Deriv.Ki[cell] -=a;

//Ito current

val=1.0f;

a = val*gtog*rN.r[cell]*rN.s[cell]*(Vshar-Ek);//Ito

Der_v += a;

Deriv.Ki[cell] -=a;

// Isus/Ikur current

val=0.00001f*(conducg2.cond_vecino4[modelo]); //Population of models

a = val*gsusg*rN.rsus[cell]*rN.ssus[cell]*(Vshar-Ek);//Isus

Der_v += a;

Deriv.Ki[cell] -=a;

//Ikr current

pi = 1.0f/(1.0f+exp((Vshar+74.0f)/24.0f));

val=1.0f;

a = val*(5.2f*powf((cKo*Kog/5.4f),0.5f))*rN.pa[cell]*pi*(Vshar-Ek);

Der_v += a;

Deriv.Ki[cell] -= a;

//Iks current

val=1.0f;

a = val*gksg*powf(rN.n[cell],2.0f)*(Vshar-Ek);

Der_v += a;

Deriv.Ki[cell] -=a;

//ICaL current

val=0.00001f*((conducg2.cond_vecino3[modelo])); //Population of models

a = val*gcalg*rN.d[cell]*rN.fca[cell]*rN.fl2[cell]*(Vshar-(60.0f+29.2f*log(Caog/1.8f)));

Der_v += a;

Deriv.Cass[cell] -= a;

//INaK current

val=0.00001f*(conducg2.cond_vecino1[modelo]); //Population of models

a = val*Inakg*((Kog*cKo)/((cKo*Kog)+KmnaKKg))*(powf(rN.Nass[cell],1.5f)/(powf(rN.Nass[cell],1.5f) + powf(KmnaKnag,1.5f))) * ((Vshar+150.0f)/(Vshar+200.0f));

Der_v += a;

Deriv.Nass[cell] -= 3.0f*a;

Deriv.Ki[cell] += 2.0f*a;

//Na-Ca channel

a = knacag*(exp(lambdag*Vshar/RTFg)*powf(rN.Nass[cell],3.0f)*Caog-exp((lambdag-1.0f)*Vshar/RTFg)*powf(Naog,3.0f)*rN.Cass[cell])/(1.0f + dNacag*(powf(Naog,3.0f)*rN.Cass[cell] + powf(rN.Nass[cell],3.0f)*Caog));

Der_v += a;

Deriv.Nass[cell] -= 3.0f*a;

Deriv.Cass[cell] += 2.0f*a;

//IbCa Current

a = gbcag*(Vshar-Eca);

Der_v += a;

Deriv.Cass[cell] -= a;

//IbNa current

a = gbnag*(Vshar-Ena);

Der_v += a;

Deriv.Nass[cell] -= a;

//IpCa current

a = Ipcag*rN.Cass[cell]/(kCapg + rN.Cass[cell]);

Der_v += a;

Deriv.Cass[cell] -= a;

//K hyperpolarization

a = gfg*rN.y[cell]*(0.2677f*(Vshar-Ena)+(1.0f-0.2677f)*(Vshar-Ek));

Der_v += a;

Deriv.Nass[cell] -= gfg*rN.y[cell]*(0.2677f*(Vshar-Ena));

Deriv.Ki[cell] -= gfg*rN.y[cell]*(1.0f-0.2677f)*(Vshar-Ek);

// IKCa current

a = 0.00001f*conducg2.cond_vecino5[modelo]*gKCag * rN.KCa[cell] * (1.0f / (1.0f + exp((Vshar - Ek + 120.0f)/45.0f))) * (Vshar - Ek);

Der_v += a;

Deriv.Ki[cell] -= a;

//Remodelling

Deriv.V[cell] = (Der_v-Stim)/(-Cmg);

//Calcium handling

V1 = 3.141592f*lcellg*(powf(1.0f*arg,2.0f))*1.0e-6f*0.5f;

V2 = 3.141592f*lcellg*(powf(2.0f*arg,2.0f)-powf(1.0f*arg,2.0f))*1.0e-6f*0.5f;

V3 = 3.141592f*lcellg*(powf(3.0f*arg,2.0f)-powf(2.0f*arg,2.0f))*1.0e-6f*0.5f;

V4 = 3.141592f*lcellg*(powf(4.0f*arg,2.0f)-powf(3.0f*arg,2.0f))*1.0e-6f*0.5f;

VSR1 = 0.0225f*V1*0.9f/1.584f;

VSR2 = 0.0225f*V2*0.9f/1.584f;

VSR3 = 0.0225f*V3*0.9f/1.584f;

VSR4 = 0.0225f*V4*0.9f/1.584f;

//Serca

k1 = 1.0e+6f*k4g;

k2 = k1*powf(kmfg,2.0f);

k3 = k4g/powf(kmrg,2.0f);

JsercaSR1 = (-k3*powf(rN.CaSR1[cell],2.0f)*(cpumpsg-rN.sercaCa1[cell])+k4g*rN.sercaCa1[cell])*V1*2.0f;

JsercaSR2 = (-k3*powf(rN.CaSR2[cell],2.0f)*(cpumpsg-rN.sercaCa2[cell])+k4g*rN.sercaCa2[cell])*V2*2.0f;

JsercaSR3 = (-k3*powf(rN.CaSR3[cell],2.0f)*(cpumpsg-rN.sercaCa3[cell])+k4g*rN.sercaCa3[cell])*V3*2.0f;

JsercaSRss = (-k3*powf(rN.CaSR4[cell],2.0f)*(cpumpsg-rN.sercaCass[cell])+k4g*rN.sercaCass[cell])*Vssg*2.0f;

J1serca = (k1*powf(rN.Cai1[cell],2.0f)*(cpumpsg-rN.sercaCa1[cell])-k2*rN.sercaCa1[cell])*V1*2.0f;

J2serca = (k1*powf(rN.Cai2[cell],2.0f)*(cpumpsg-rN.sercaCa2[cell])-k2*rN.sercaCa2[cell])*V2*2.0f;

J3serca = (k1*powf(rN.Cai3[cell],2.0f)*(cpumpsg-rN.sercaCa3[cell])-k2*rN.sercaCa3[cell])*V3*2.0f;

Jssserca = (k1*powf(rN.Cass[cell],2.0f)*(cpumpsg-rN.sercaCass[cell])-k2*rN.sercaCass[cell])*Vssg*2.0f;

Deriv.sercaCa1[cell] = 0.5f*(J1serca-JsercaSR1)/V1;

Deriv.sercaCa2[cell] = 0.5f*(J2serca-JsercaSR2)/V2;

Deriv.sercaCa3[cell] = 0.5f*(J3serca-JsercaSR3)/V3;

Deriv.sercaCass[cell] = 0.5f*(Jssserca-JsercaSRss)/Vssg;

//RyR

RyRsrCa1 = 1.0f - 1.0f/(1.0f+exp((rN.CaSR1[cell]-0.3f/2.0f)/0.1f));

RyRsrCa2 = 1.0f - 1.0f/(1.0f+exp((rN.CaSR2[cell]-0.3f/2.0f)/0.1f));

RyRsrCa3 = 1.0f - 1.0f/(1.0f+exp((rN.CaSR3[cell]-0.3f/2.0f)/0.1f));

RyRsrCass = 1.0f - 1.0f/(1.0f+exp((rN.CaSR4[cell]-0.3f/2.0f)/0.1f));

Jrel1 = 1.6f/1.584f*rN.RyRo1[cell]*rN.RyRc1[cell]*RyRsrCa1*(rN.CaSR1[cell]-rN.Cai1[cell])*V1;

Jrel2 = 1.6f/1.584f*rN.RyRo2[cell]*rN.RyRc2[cell]*RyRsrCa2*(rN.CaSR2[cell]-rN.Cai2[cell])*V2;

Jrel3 = 1.6f/1.584f*rN.RyRo3[cell]*rN.RyRc3[cell]*RyRsrCa3*(rN.CaSR3[cell]-rN.Cai3[cell])*V3;

Jrelss =900.0f/1.584f*rN.RyRoss[cell]*rN.RyRcss[cell]*RyRsrCass*(rN.CaSR4[cell]-rN.Cass[cell])*Vssg; // 625.0f*rN.RyRoss[cell]*rN.RyRcss[cell]*RyRsrCass*(rN.CaSR4[cell]-rN.Cass[cell])*Vssg;

//SR calcium leak

Jsrleak1 = ksrleakg*(rN.CaSR1[cell]-rN.Cai1[cell])*V1/1.584f;

Jsrleak2 = ksrleakg*(rN.CaSR2[cell]-rN.Cai2[cell])*V2/1.584f;

Jsrleak3 = ksrleakg*(rN.CaSR3[cell]-rN.Cai3[cell])*V3/1.584f;

Jsrleakss = ksrleakg*(rN.CaSR4[cell]-rN.Cass[cell])*Vssg/1.584f;

/* Sarcoplasmis reticulum*/

Jj_nj = DCag*((3.141592f*rjunctg*lcellg)/(0.01f*1.2f + arg/2.0f))*(rN.Cass[cell]-rN.Cai4[cell])*1.0e-6f; //ojo que 1e-6 no salia en las formulas

JCa1 = -J1serca+Jsrleak1+Jrel1;

JCa2 = -J2serca+Jsrleak2+Jrel2;

JCa3 = -J3serca+Jsrleak3+Jrel3;

JCa4 = Jj_nj;

JCass = -Jj_nj+Jsrleakss-Jssserca+Jrelss;

JSRCa1 = JsercaSR1-Jsrleak1-Jrel1;

JSRCa2 = JsercaSR2-Jsrleak1-Jrel2;

JSRCa3 = JsercaSR3-Jsrleak1-Jrel3;

JSRCa4 = JsercaSRss-Jsrleakss-Jrelss;

JNa = DNag * ((3.141592f*rjunctg*lcellg)/(0.01f*1.2f + 2.0f*arg))*(rN.Nass[cell]-rN.Nai[cell])*1.0e-6f; //ojo que 1e-6 no salia en las formulas

betaNass = 1.0f/(1.0f+(KdBNag*BNag/powf(rN.Nass[cell]+KdBNag,2.0f)));

// Calcium buffer

betass = 1.0f/(1.0f+(KdSLlowg*SLlowg/powf(rN.Cass[cell]+KdSLlowg,2.0f))+(KdSLhighg*SLhighg/powf(rN.Cass[cell]+KdSLhighg,2.0f))+(KdBCag*BCag/powf(rN.Cass[cell]+KdBCag,2.0f)));

betai1 = 1.0f/(1.0f+(KdBCag*BCag/powf(rN.Cai1[cell]+KdBCag,2.0f)));

betai2 = 1.0f/(1.0f+(KdBCag*BCag/powf(rN.Cai2[cell]+KdBCag,2.0f)));

betai3 = 1.0f/(1.0f+(KdBCag*BCag/powf(rN.Cai3[cell]+KdBCag,2.0f)));

betai4 = 1.0f/(1.0f+(KdBCag*BCag/powf(rN.Cai4[cell]+KdBCag,2.0f)));

gammai1 = KdBCag*BCag/powf(rN.Cai1[cell]+KdBCag,2.0f);

gammai2 = KdBCag*BCag/powf(rN.Cai2[cell]+KdBCag,2.0f);

gammai3 = KdBCag*BCag/powf(rN.Cai3[cell]+KdBCag,2.0f);

gammai4 = KdBCag*BCag/powf(rN.Cai4[cell]+KdBCag,2.0f);

betaSR1 = 1.0f/(1.0f+(KdCSQNg*CSQNg/powf(rN.CaSR1[cell]+KdCSQNg,2.0f)));

betaSR2 = 1.0f/(1.0f+(KdCSQNg*CSQNg/powf(rN.CaSR2[cell]+KdCSQNg,2.0f)));

betaSR3 = 1.0f/(1.0f+(KdCSQNg*CSQNg/powf(rN.CaSR3[cell]+KdCSQNg,2.0f)));

betaSR4 = 1.0f/(1.0f+(KdCSQNg*CSQNg/powf(rN.CaSR4[cell]+KdCSQNg,2.0f)));

//Intra sarcoplasmic reticulum calcium

Deriv.Cass[cell] = betass*(JCass/Vssg + Deriv.Cass[cell]/(2.0f*Vssg*Fg));

Deriv.Cai1[cell] = betai1*(DCag+gammai1*DCaBmg)*((rN.Cai2[cell]-rN.Cai1[cell])/powf(arg,2.0f) + (rN.Cai2[cell]-rN.Cai1[cell])/(2.0f*1.0f*powf(arg,2.0f)))-(2.0f*betai1*gammai1*DCaBmg/(KdBCag+rN.Cai1[cell]))*powf((rN.Cai2[cell]-rN.Cai1[cell])/(2.0f*arg),2.0f)+betai1*JCa1/V1;

Deriv.Cai2[cell] = betai2*(DCag+gammai2*DCaBmg)*((rN.Cai3[cell]-2.0f*rN.Cai2[cell]+rN.Cai1[cell])/powf(arg,2.0f) + (rN.Cai3[cell]-rN.Cai1[cell])/(2.0f*2.0f*powf(arg,2.0f)))-(2.0f*betai2*gammai2*DCaBmg/(KdBCag+rN.Cai2[cell]))*powf((rN.Cai3[cell]-rN.Cai1[cell])/(2.0f*arg),2.0f)+betai2*JCa2/V2;

Deriv.Cai3[cell] = betai3*(DCag+gammai3*DCaBmg)*((rN.Cai4[cell]-2.0f*rN.Cai3[cell]+rN.Cai2[cell])/powf(arg,2.0f) + (rN.Cai4[cell]-rN.Cai2[cell])/(2.0f*3.0f*powf(arg,2.0f)))-(2.0f*betai3*gammai3*DCaBmg/(KdBCag+rN.Cai3[cell]))*powf((rN.Cai4[cell]-rN.Cai2[cell])/(2.0f*arg),2.0f)+betai3*JCa3/V3;

Deriv.Cai4[cell] = betai4*(DCag+gammai4*DCaBmg)*((-rN.Cai4[cell]+rN.Cai3[cell])/powf(arg,2.0f) + (rN.Cai4[cell]-rN.Cai3[cell])/(2.0f*4.0f*powf(arg,2.0f)))-(2.0f*betai4*gammai4*DCaBmg/(KdBCag+rN.Cai4[cell]))*powf((rN.Cai4[cell]-rN.Cai3[cell])/(2.0f*arg),2.0f)+betai4*JCa4/V4;

Deriv.CaSR1[cell] = betaSR1*DCaSRg*((rN.CaSR2[cell]-rN.CaSR1[cell])/powf(arg,2.0f) + (rN.CaSR2[cell]-rN.CaSR1[cell])/(2.0f*1.0f*powf(arg,2.0f)))+betaSR1*JSRCa1/VSR1;

Deriv.CaSR2[cell] = betaSR2*DCaSRg*((rN.CaSR3[cell]-2.0f*rN.CaSR2[cell]+rN.CaSR1[cell])/powf(arg,2.0f) + (rN.CaSR3[cell]-rN.CaSR1[cell])/(2.0f*2.0f*powf(arg,2.0f)))+betaSR2*JSRCa2/VSR2;

Deriv.CaSR3[cell] = betaSR3*DCaSRg*((rN.CaSR4[cell]-2.0f*rN.CaSR3[cell]+rN.CaSR2[cell])/powf(arg,2.0f) + (rN.CaSR4[cell]-rN.CaSR2[cell])/(2.0f*3.0f*powf(arg,2.0f)))+betaSR3*JSRCa3/VSR3;

Deriv.CaSR4[cell] = betaSR4*DCaSRg*((-rN.CaSR4[cell]+rN.CaSR3[cell])/powf(arg,2.0f) + (rN.CaSR4[cell]-rN.CaSR3[cell])/(2.0f*4.0f*powf(arg,2.0f)))+betaSR4*JSRCa4/VSR4;

//Concentrations

Deriv.Nass[cell] = betaNass*(-JNa/Vssg + Deriv.Nass[cell]/(Vssg*Fg));

Deriv.KCa[cell] = (1.0f- rN.KCa[cell])*47.0e6f*(powf(rN.Cass[cell],2.0f))-rN.KCa[cell]*13.0f;

Deriv.Nai[cell] = JNa/(V1+V2+V3+V4);

Deriv.Ki[cell] = (Deriv.Ki[cell])/((V1+V2+V3+V4+Vssg)*Fg);

}else{

//Fibroblast model

float EK_fb,ENa_fb,IKv,tau_rKv,tau_sKv,rKv_inf,sKv_inf;

float alfaK1,betaK1,IK1fb,INakfb,IbNa;

float gK1_fb = 0.4822f;

float V_rev = -150.0f;

float B = -200.0f;

float kmK = 1.0f;

float kmNa = 11.0f;

float INak_max = 1.644f;

float gbNa = 0.0095f; //nS/pF

float Ko_fb = 5.3581f;

float Nao_fb = 130.0110f;

float gKv = 0.25f; //nS/pF

float Cm_fb = 0.0063f; //nF

float Vss_fb = 0.00137f; //nL

float ggap = 0.5f; //nS/pF

EK_fb = RTFg/1000*log(Ko_fb/rN.Ki[cell]);

ENa_fb = RTFg/1000*log(Nao_fb/rN.Nai[cell]);

// Currents of fibroblast

// IKv

IKv = gKv*rN.pa[cell]*rN.n[cell]*(rN.V[cell]- EK_fb);

Der_v +=IKv;

tau_rKv = 0.0203f + 0.1380f*exp(powf((-(rN.V[cell]+20.0f)/25.9f),2.0f));

tau_sKv = 1.574f + 5.268f*exp(powf(-((rN.V[cell]+23.0f)/22.7f),2.0f));

// Active model

rKv_inf = 1.0f/(1.0f+exp(-(rN.V[cell]+20.0f)/11.0f));

sKv_inf = 1.0f/(1.0f+exp((rN.V[cell]+23.0f)/7.0f));

// IK1_fb

alfaK1 = 0.1f/(1.0f + exp(0.06f*(rN.V[cell]- EK_fb-200.0f)));

betaK1 = (3.0f*exp(0.0002f*(rN.V[cell]- EK_fb + 100.0f)) + exp(0.1f*(rN.V[cell]- EK_fb - 10.0f)))/(1.0f + exp(-0.5f*(rN.V[cell]- EK_fb)));

IK1fb = gK1_fb*((alfaK1/(alfaK1 + betaK1))*(rN.V[cell]-EK_fb));

Der_v += IK1fb;

// INak

INakfb = INak_max*(Ko_fb/(Ko_fb + kmK))*powf((rN.Nai[cell]/(rN.Nai[cell]+kmNa)),1.5f)*((rN.V[cell]-V_rev)/(rN.V[cell]-B));

Der_v += INakfb;

// IbNa

IbNa = gbNa*(rN.V[cell]- ENa_fb);

Der_v += IbNa;

// Igap

EKgap = R*T/F * log( Kifb / Ki);

ENagap = R*T/F * log( Naifb / Nass);

Igapk = ggapk * ((y(i_V)-y(i_Vfb))-EKgap);

IgapNa = ggapNa * ((y(i_V)-y(i_Vfb))-ENagap);

Igap = Igapk + IgapNa;

Igap = ggap * (y(i_V) - rN.V[cell]);

Der_v += Igap;

// Ifb

//Ifb = IKv + IK1fb + INakfb + IbNa;

//dy

Deriv.V[cell]= ((Der_v-Stim)/-Cm_fb); // currents are in (pA)

// IKv

Deriv.pa[cell] = (rKv_inf - rN.pa[cell])/tau_rKv;

Deriv.n[cell]= (sKv_inf - rN.n[cell])/tau_sKv;

// Intracellular Ionic Concentrations

Deriv.Ki[cell] = -(IK1fb + IKv -2.0f*INakfb)/(Vss_fb*Fg);

Deriv.Nai[cell] = -(IbNa + 3.0f*INakfb)/(Vss_fb*Fg);

}

}// of Total_transmembrane_current

__device__ void quita_casos(state_variables &estado, int cell)

{

// Esto es para evitar divisiones por cero

int cond=0;

float Vshar=estado.V[cell];

cond=abs(Vshar)>1e-4f;

estado.V[cell]=estado.V[cell]+(1-cond)*(1e-3f);

}

__device__ void copia(state_variables &estadoin,state_variables &estadoout, int cell)

{

estadoout.V[cell] = estadoin.V[cell];

estadoout.Nai[cell] = estadoin.Nai[cell];

estadoout.Ki[cell] = estadoin.Ki[cell];

estadoout.sercaCa1[cell] = estadoin.sercaCa1[cell];

estadoout.sercaCa2[cell] = estadoin.sercaCa2[cell];

estadoout.sercaCa3[cell] = estadoin.sercaCa3[cell];

estadoout.sercaCass[cell] = estadoin.sercaCass[cell];

estadoout.Cass[cell] = estadoin.Cass[cell];

estadoout.Cai1[cell] = estadoin.Cai1[cell];

estadoout.Cai2[cell] = estadoin.Cai2[cell];

estadoout.Cai3[cell] = estadoin.Cai3[cell];

estadoout.Cai4[cell] = estadoin.Cai4[cell];

estadoout.CaSR1[cell] = estadoin.CaSR1[cell];

estadoout.CaSR2[cell] = estadoin.CaSR2[cell];

estadoout.CaSR3[cell] = estadoin.CaSR3[cell];

estadoout.CaSR4[cell] = estadoin.CaSR4[cell];

estadoout.Nass[cell] = estadoin.Nass[cell];

estadoout.KCa[cell] = estadoin.KCa[cell];

//estadoout.Cai[cell] = estadoin.Cai[cell];

estadoout.m[cell] = estadoin.m[cell];

estadoout.h1[cell] = estadoin.h1[cell];

estadoout.h2[cell] = estadoin.h2[cell];

estadoout.d[cell] = estadoin.d[cell];

estadoout.fl1[cell] = estadoin.fl1[cell];

estadoout.fl2[cell] = estadoin.fl2[cell];

estadoout.fca[cell] = estadoin.fca[cell];

estadoout.r[cell] = estadoin.r[cell];

estadoout.s[cell] = estadoin.s[cell];

estadoout.rsus[cell] = estadoin.rsus[cell];

estadoout.ssus[cell] = estadoin.ssus[cell];

estadoout.n[cell] = estadoin.n[cell];

estadoout.pa[cell] = estadoin.pa[cell];

estadoout.y[cell] = estadoin.y[cell];

estadoout.RyRoss[cell] = estadoin.RyRoss[cell];

estadoout.RyRcss[cell] = estadoin.RyRcss[cell];

estadoout.RyRass[cell] = estadoin.RyRass[cell];

estadoout.RyRo1[cell] = estadoin.RyRo1[cell];

estadoout.RyRc1[cell] = estadoin.RyRc1[cell];

estadoout.RyRa1[cell] = estadoin.RyRa1[cell];

estadoout.RyRo2[cell] = estadoin.RyRo2[cell];

estadoout.RyRc2[cell] = estadoin.RyRc2[cell];

estadoout.RyRa2[cell] = estadoin.RyRa2[cell];

estadoout.RyRo3[cell] = estadoin.RyRo3[cell];

estadoout.RyRc3[cell] = estadoin.RyRc3[cell];

estadoout.RyRa3[cell] = estadoin.RyRa3[cell];

// estadoout.over[cell] = estadoin.over[cell];

}

__device__ void gating_vars_rl_explicit(state_variables &rNant, state_variables &out, float tim,float inct,int cell)

{

Vshar=rNant.V[cell];

if (tipo_celg.tipo[cell]==0){

float tau_rKv,tau_sKv,rKv_inf,sKv_inf;

tau_rKv = 0.0203f + 0.1380f*exp(powf(-(rNant.V[cell]+20.0f)/25.9f,2.0f));

tau_sKv = 1.574f + 5.268f*exp(powf(-(rNant.V[cell]+23.0f)/22.7f,2.0f));

rKv_inf = 1.0f/(1.0f+exp(-(rNant.V[cell]+20.0f)/11.0f));

sKv_inf = 1.0f/(1.0f+exp((rNant.V[cell]+23.0f)/7.0f));

out.pa[cell] = rKv_inf+(rNant.pa[cell]-rKv_inf)*exp(-inct/tau_rKv);

out.n[cell]= sKv_inf+(rNant.n[cell]-sKv_inf)*exp(-inct/tau_sKv);

}
