# Supplementary figures and images for: Artificial intelligence analysis of the impact of fibrosis in arrhythmogenesis and drug response
Source: Front Physiol. 2022 Oct 12;13:1025430. doi: 10.3389/fphys.2022.1025430 (PMC9596790; doi:10.3389/fphys.2022.1025430)

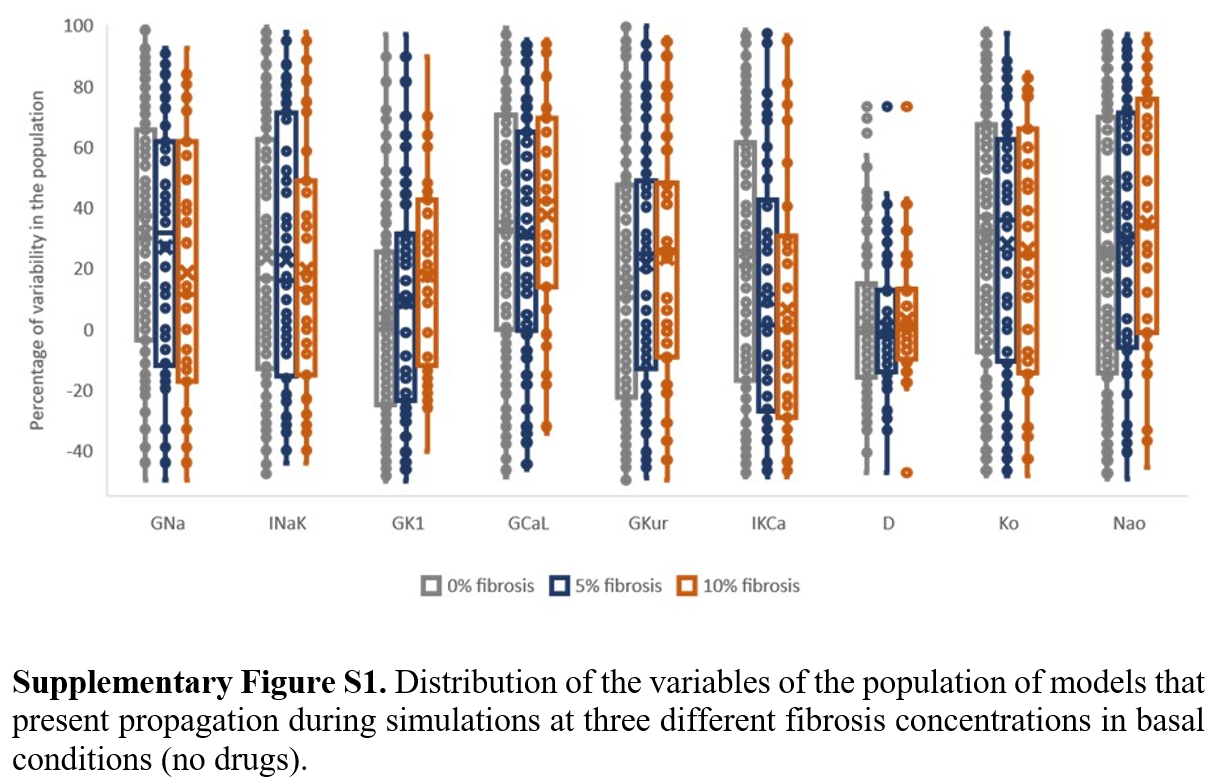

Supplement: Supplementary file 5 [file Image1.png]
