# Supplementary material for: Artificial intelligence analysis of the impact of fibrosis in arrhythmogenesis and drug response
Source: Front Physiol. 2022 Oct 12;13:1025430. doi: 10.3389/fphys.2022.1025430 (PMC9596790; doi:10.3389/fphys.2022.1025430)
Supplement: Supplementary file 6 [file DataSheet1.DOCX]

float Cm = 0.05f*1.32f; /*nF*/

//S1-S2 stimuli

//S1

const float S1_amp=0.0f; //-100*Cm; //Amplitude

const float S1_total_time = 0.003f; // duration

float S1_begin = 0.3f;

float S1_period = 1.0f; // period

//S2

const float S2_amp=4000.0f; //-100*Cm; //Amplitude

const float S2_total_time = 0.003f; // duration

float S2_begin = 0.1f;

float S2_period=1.0f,

const int pasos_envio=20000;

const int interv_envio=1/(1000*dt); //Sampling frequency

const int pasos_guardar=1; //Saving time

//constantes (inc Cm)

float gto = 4.18f;

float kCa = 0.7e-3;

float gcal = 2.925f;

float gcal2 = 2.925f;

float gk1 = 4.704f;

float gkr = 5.2f;

float gkr2 = 5.2f;

float gsus =1.395f;

float gks = 0.42875f;

float gbna = 0.060599f;

float gbca = 0.078f;

float gf = 1.0f;

float gKCa = 1.8f;

float gna = 524.8f;

float glna = 0.351f;

float Ipca = 2.0f;

float Inak = 70.8253f;

float knaca = 0.01215f;

float R = 8314.0f;

float T = 310.15f;

float F = 96487.0f;

float RTF = 26.72f;

float Vi = 0.00814997f;

float FVi = (F*Vi);

float Vc = 0.136f*Vi;

float Vss = 4.99232e-5f*1.584f;

float Ko = 5.4f;

float Nao = 130.0f;

float Cao = 1.8f;

float lambda = 0.45f;

float KmnaKK = 1.0f;

float KmnaKna = 11.0f;

float dNaca = 0.0003f;

float kCap = 0.0005f;

float rjunct = 6.5f*1.2f;

float lcell = 134.2561f;

float ar = 1.625f*1.2f;

float DCa = 833.0f;

float DCaBm = 28.8f;

float DCaSR = 50.7f;

float BCa = 0.024f;

float SLlow = 165.0f;

float SLhigh = 13.0f;

float KdBCa = 0.00238f;

float KdSLlow = 1.1f;

float KdSLhigh = 0.013f;

float CSQN = 6.7f;

float KdCSQN = 0.8f;

float DNa = 0.146f;

float BNa = 1.1319f;

float KdBNa = 10.0f;

float kmf = 0.00029688f;

float kmr = 2.324f;

float k4 =17.0f;

float cpumps =0.0159f;

float ksrleak = 0.006f;

// GPU configuration

__constant__ float Cmg; /*pF*/

__constant__ float Rg; /*J/mol*K*/

__constant__ float Tg; /*K*/

__constant__ float Fg; /*C/mmol*/

__constant__ float RTFg; /* A widely used combination */

__constant__ float Vig; /*um^3*/

__constant__ float FVig; /* A widely used combination */

__constant__ float Vcg;

__constant__ float Vssg;

__constant__ float Kog; /*mM*/

__constant__ float Naog; /*mM*/

__constant__ float Caog; /*mM*/

__constant__ float gnag; /*nS/pF*/

__constant__ float glnag; /*nS/pF*/

__constant__ float gk1g; /*nS/pF*/

__constant__ float gtog; /*nS/pF*/

__constant__ float gkrg; /*nS/pF*/

__constant__ float gkr2g; /*nS/pF*/

__constant__ float gksg; /*nS/pF*/

__constant__ float kCag; /*nS/pF*/

__constant__ float gcalg; /*nS/pF*/

__constant__ float gcal2g; /*nS/pF*/

__constant__ float gbcag; /*nS/pF*/

__constant__ float gbnag; /*nS/pF*/

__constant__ float gsusg; /*nS/pF*/

__constant__ float gfg;

__constant__ float gKCag; /*nS/pF*/

__constant__ float Ipcag; /*pA/pF*/

__constant__ float Inakg; /*pA/pF*/

__constant__ float knacag; /*pA/pF*/ /*pA/pF*/

__constant__ float lambdag;

__constant__ float KmnaKKg;

__constant__ float KmnaKnag;

__constant__ float dNacag;

__constant__ float kCapg;

__constant__ float rjunctg; /*um*/

__constant__ float lcellg; /*um*/

__constant__ float arg; /*um*/

__constant__ float DCag; /*mm/s*/

__constant__ float DCaBmg; /*mm/s*/

__constant__ float DCaSRg; /*mm/s*/

__constant__ float BCag; /*mM*/

__constant__ float SLlowg; /*mM*/

__constant__ float SLhighg; /*mM*/

__constant__ float KdBCag; /*mM*/

__constant__ float KdSLlowg; /*mM*/

__constant__ float KdSLhighg; /*mM*/

__constant__ float CSQNg; /*mM Shannon*/

__constant__ float KdCSQNg; /*mM Shannon*/

__constant__ float DNag;

__constant__ float BNag;

__constant__ float KdBNag;

__constant__ float kmfg; /*mM*/

//__constant__ float kmfremg; /*mM*/

__constant__ float kmrg; /*mM*/

//__constant__ float kmrremg; /*mM*/

__constant__ float k4g; /*seg-1*/

__constant__ float cpumpsg; /*mM*/

__constant__ float ksrleakg; /*seg-1*/

// __constant__ float Verg ;

void copia_parametros_s__gpu()

{

float *tempf = (float*)malloc(sizeof(float));

tempf[0]=Cm;

cudaMemcpyToSymbol(Cmg, tempf, sizeof(float)) ;

tempf[0]=R;

cudaMemcpyToSymbol(Rg, tempf, sizeof(float)) ;

tempf[0]=T;

cudaMemcpyToSymbol(Tg, tempf, sizeof(float)) ;

tempf[0]=F;

cudaMemcpyToSymbol(Fg, tempf, sizeof(float)) ;

tempf[0]=(R*T/F);

cudaMemcpyToSymbol(RTFg, tempf, sizeof(float)) ;

tempf[0]=Vi;

cudaMemcpyToSymbol(Vig, tempf, sizeof(float)) ;

tempf[0]=(F*Vi);

cudaMemcpyToSymbol(FVig, tempf, sizeof(float));

tempf[0]=Vc;

cudaMemcpyToSymbol(Vcg, tempf, sizeof(float));

tempf[0]=Vss;

cudaMemcpyToSymbol(Vssg, tempf, sizeof(float));

tempf[0]=Ko;

cudaMemcpyToSymbol(Kog, tempf, sizeof(float)) ;

tempf[0]=Nao;

cudaMemcpyToSymbol(Naog, tempf, sizeof(float));

tempf[0]=Cao;

cudaMemcpyToSymbol(Caog, tempf, sizeof(float));

tempf[0]=gna;

cudaMemcpyToSymbol(gnag, tempf, sizeof(float)) ;

tempf[0]=glna;

cudaMemcpyToSymbol(glnag, tempf, sizeof(float)) ;

tempf[0]=gk1;

cudaMemcpyToSymbol(gk1g, tempf, sizeof(float)) ;

tempf[0]=gto;

cudaMemcpyToSymbol(gtog, tempf, sizeof(float)) ;

tempf[0]=gkr;

cudaMemcpyToSymbol(gkrg, tempf, sizeof(float)) ;

tempf[0]=gkr2;

cudaMemcpyToSymbol(gkr2g, tempf, sizeof(float)) ;

tempf[0]=gks;

cudaMemcpyToSymbol(gksg, tempf, sizeof(float)) ;

tempf[0]=kCa;

cudaMemcpyToSymbol(gksg, tempf, sizeof(float)) ;

tempf[0]=gcal;

cudaMemcpyToSymbol(gcalg, tempf, sizeof(float)) ;

tempf[0]=gcal2;

cudaMemcpyToSymbol(gcal2g, tempf, sizeof(float)) ;

tempf[0]=gbca;

cudaMemcpyToSymbol(gbcag, tempf, sizeof(float)) ;

tempf[0]=gbna;

cudaMemcpyToSymbol(gbnag, tempf, sizeof(float)) ;

tempf[0]=gsus;

cudaMemcpyToSymbol(gsusg, tempf, sizeof(float)) ;

tempf[0]=gf;

cudaMemcpyToSymbol(gfg, tempf, sizeof(float)) ;

tempf[0]=gKCa; //INCLUIDA

cudaMemcpyToSymbol(gKCag, tempf, sizeof(float)) ;

tempf[0]=Inak;

cudaMemcpyToSymbol(Inakg, tempf, sizeof(float)) ;

tempf[0]=knaca;

cudaMemcpyToSymbol(knacag, tempf, sizeof(float)) ;

tempf[0]=Ipca;

cudaMemcpyToSymbol(Ipcag, tempf, sizeof(float) );

tempf[0]=lambda;

cudaMemcpyToSymbol(lambdag, tempf, sizeof(float)) ;

tempf[0]=KmnaKK;

cudaMemcpyToSymbol(KmnaKKg, tempf, sizeof(float)) ;

tempf[0]=KmnaKna;

cudaMemcpyToSymbol(KmnaKnag, tempf, sizeof(float)) ;

tempf[0]=dNaca;

cudaMemcpyToSymbol(dNacag, tempf, sizeof(float)) ;

tempf[0]=kCap;

cudaMemcpyToSymbol(kCapg, tempf, sizeof(float)) ;

tempf[0]=rjunct;

cudaMemcpyToSymbol(rjunctg, tempf, sizeof(float)) ;

tempf[0]=lcell;

cudaMemcpyToSymbol(lcellg, tempf, sizeof(float)) ;

tempf[0]=ar;

cudaMemcpyToSymbol(arg, tempf, sizeof(float)) ;

tempf[0]=DCa;

cudaMemcpyToSymbol(DCag, tempf, sizeof(float)) ;

tempf[0]=DCaBm;

cudaMemcpyToSymbol(DCaBmg, tempf, sizeof(float)) ;

tempf[0]=DCaSR;

cudaMemcpyToSymbol(DCaSRg, tempf, sizeof(float)) ;

tempf[0]=BCa;

cudaMemcpyToSymbol(BCag, tempf, sizeof(float)) ;

tempf[0]=SLlow;

cudaMemcpyToSymbol(SLlowg, tempf, sizeof(float)) ;

tempf[0]=SLhigh;

cudaMemcpyToSymbol(SLhighg, tempf, sizeof(float)) ;

tempf[0]=KdBCa;

cudaMemcpyToSymbol(KdBCag, tempf, sizeof(float)) ;

tempf[0]=KdSLlow;

cudaMemcpyToSymbol(KdSLlowg, tempf, sizeof(float)) ;

tempf[0]=KdSLhigh;

cudaMemcpyToSymbol(KdSLhighg, tempf, sizeof(float)) ;

tempf[0]=CSQN;

cudaMemcpyToSymbol(CSQNg, tempf, sizeof(float)) ;

tempf[0]=KdCSQN;

cudaMemcpyToSymbol(KdCSQNg, tempf, sizeof(float)) ;

tempf[0]=DNa;

cudaMemcpyToSymbol(DNag, tempf, sizeof(float)) ;

tempf[0]=BNa;

cudaMemcpyToSymbol(BNag, tempf, sizeof(float)) ;

tempf[0]=KdBNa;

cudaMemcpyToSymbol(KdBNag, tempf, sizeof(float)) ;

tempf[0]=kmf;

cudaMemcpyToSymbol(kmfg, tempf, sizeof(float)) ;

/*

tempf[0]=kmfrem;

cudaMemcpyToSymbol(kmfremg, tempf, sizeof(float)) ;

*/

tempf[0]=kmr;

cudaMemcpyToSymbol(kmrg, tempf, sizeof(float)) ;

/*

tempf[0]=kmrrem;

cudaMemcpyToSymbol(kmrremg, tempf, sizeof(float)) ;

*/

tempf[0]=k4;

cudaMemcpyToSymbol(k4g, tempf, sizeof(float)) ;

tempf[0]=cpumps;

cudaMemcpyToSymbol(cpumpsg, tempf, sizeof(float)) ;

tempf[0]=ksrleak;

cudaMemcpyToSymbol(ksrleakg, tempf, sizeof(float)) ;

// tempf[0]=Ver;

// cudaMemcpyToSymbol(Verg, tempf, sizeof(float)) ;

free(tempf);

}

//Variables y constantes en GPU de Estimulaci�n y envio de dat

//***********STIMULATION PARAMETERS****************S1

__constant__ float S1_ampg;

__constant__ float S1_total_timeg;

__constant__ float S1_beging;

__constant__ float S1_periodg;

//***********STIMULATION PARAMETERS****************S2

__constant__ float S2_ampg;

__constant__ float S2_total_timeg;

__constant__ float S2_beging;

__constant__ float S2_periodg;

__constant__ int pasos_enviog;

__constant__ int interv_enviog;

__constant__ int pasos_guardarg;

__constant__ float powg;

__constant__ float alphag;

__constant__ float dg;

__constant__ float ggg;

__constant__ float c1g;

__constant__ float c2g;

__constant__ float c3g;

__constant__ float p31g;

__constant__ float p32g;

__constant__ float p33g;

float power=1.0f/3.0f;

float alpha =2.0f-sqrt(2.0f);

float d=alpha/2.0f;

float gg=sqrt(2.0f)/4.0f;

float c1=(alpha -1.0f)/3.0f;

float c2=1.0f/3.0f;

float c3= -alpha/3.0f;

float p31=1.5f +sqrt(2.0f);

float p32=2.5f +2.0f*sqrt(2.0f);

float p33=-(6.0f+4.5f*sqrt(2.0f));

void copia_parametros_gpu()

{

//

float *tempf = (float*)malloc(sizeof(float));

//Estimulacion S1

tempf[0]=S1_amp;

//cudaMemcpyToSymbol(S1_amp,tempf,sizeof(float));

cudaMemcpyToSymbol(S1_ampg, tempf, sizeof(float)) ;

tempf[0]=S1_total_time;

cudaMemcpyToSymbol(S1_total_timeg, tempf, sizeof(float)) ;

tempf[0]=S1_begin;

cudaMemcpyToSymbol(S1_beging, tempf, sizeof(float)) ;

tempf[0]=S1_period;

cudaMemcpyToSymbol(S1_periodg, tempf, sizeof(float)) ;

//Estimulacion S2

tempf[0]=S2_amp;

cudaMemcpyToSymbol(S2_ampg, tempf, sizeof(float)) ;

tempf[0]=S2_total_time;

cudaMemcpyToSymbol(S2_total_timeg, tempf, sizeof(float)) ;

tempf[0]=S2_begin;

cudaMemcpyToSymbol(S2_beging, tempf, sizeof(float)) ;

tempf[0]=S2_period;

cudaMemcpyToSymbol(S2_periodg, tempf, sizeof(float)) ;

//Conductancia y parametros de envio

// tempf[0]=Cond;

// cudaMemcpyToSymbol(Condg, tempf, sizeof(float)) ;

int *tempi = (int*)malloc(sizeof(int));

tempi[0]=pasos_envio;

cudaMemcpyToSymbol(pasos_enviog, tempi, sizeof(int)) ;

tempi[0]=interv_envio;

cudaMemcpyToSymbol(interv_enviog, tempi, sizeof(int)) ;

tempi[0]=pasos_guardar;

cudaMemcpyToSymbol(pasos_guardarg, tempi, sizeof(int)) ;

// Parametros metodos TR, BDF2 y predictores y estimador de error

tempf[0]=power;

cudaMemcpyToSymbol(powg, tempf, sizeof(float)) ;

tempf[0]=alpha;

cudaMemcpyToSymbol(alphag, tempf, sizeof(float)) ;

tempf[0]=d;

cudaMemcpyToSymbol(dg, tempf, sizeof(float)) ;

tempf[0]=gg;

cudaMemcpyToSymbol(ggg, tempf, sizeof(float)) ;

tempf[0]=c1;

cudaMemcpyToSymbol(c1g, tempf, sizeof(float)) ;

tempf[0]=c2;

cudaMemcpyToSymbol(c2g, tempf, sizeof(float)) ;

tempf[0]=c3;

cudaMemcpyToSymbol(c3g, tempf, sizeof(float)) ;

tempf[0]=p31;

cudaMemcpyToSymbol(p31g, tempf, sizeof(float)) ;

tempf[0]=p32;

cudaMemcpyToSymbol(p32g, tempf, sizeof(float)) ;

tempf[0]=p33;

cudaMemcpyToSymbol(p33g, tempf, sizeof(float)) ;

free(tempf);

free(tempi);

}
